# The Basal Nodosaurid Ankylosaur *Europelta carbonensis* n. gen., n. sp. from the Lower Cretaceous (Lower Albian) Escucha Formation of Northeastern Spain

**DOI:** 10.1371/journal.pone.0080405

**Published:** 2013-12-02

**Authors:** James I. Kirkland, Luis Alcalá, Mark A. Loewen, Eduardo Espílez, Luis Mampel, Jelle P. Wiersma

**Affiliations:** 1 Utah Geological Survey, Salt Lake City, Utah, United States of America; 2 Fundación Conjunto Paleontológico de Teruel-Dinópolis, Museo Aragonés de Paleontología, Teruel, Spain; 3 Natural History Museum of Utah, Salt Lake City, Utah, United States of America; 4 Department of Geology & Geophysics, University of Utah, Salt Lake City, Utah, United States of America; University of Birmingham, United Kingdom

## Abstract

Nodosaurids are poorly known from the Lower Cretaceous of Europe. Two associated ankylosaur skeletons excavated from the lower Albian carbonaceous member of the Escucha Formation near Ariño in northeastern Teruel, Spain reveal nearly all the diagnostic recognized character that define nodosaurid ankylosaurs. These new specimens comprise a new genus and species of nodosaurid ankylosaur and represent the single most complete taxon of ankylosaur from the Cretaceous of Europe. These two specimens were examined and compared to all other known ankylosaurs. Comparisons of these specimens document that *Europelta carbonensis* n. gen., n. sp. is a nodosaur and is the sister taxon to the Late Cretaceous nodosaurids *Anoplosaurus*, *Hungarosaurus*, and *Struthiosaurus*, defining a monophyletic clade of European nodosaurids– the Struthiosaurinae.

## Introduction

Ankylosaurs were first described from the Lower Cretaceous of England with *Hylaeosaurus armatus* (Valanginian) described in 1833 [1]–[3]. *Hylaeosaurus* is one of the three dinosaurs on which the Dinosauria were defined [4] and one of the first dinosaurs for which a full-sized life reconstruction was attempted at the Crystal Palace Park in London in 1854 [5]. Although first mentioned in an anonymous article in the September 16^th^ 1865 issue of the “The Illustrated London News” by Sir Richard Owen [6], the Early Cretaceous (Barremian) *Polacanthus* was not described formally as *Polacanthus foxii* by Hulke until 1882 [7]–[10]. The abundant plates and spines of these ankylosaurs are characteristic of the Lower Cretaceous up into the lower part of the Aptian stage [11], [12]. In 1867, Huxley described the fragmentary *Acanthopholis* from the base of the Upper Cretaceous (Cenomanian) [13]–[15]. Additionally, in 1879, Seeley [16] described the juvenile nodosaurid *Anoplosaurus curtonotus* [17] from the uppermost Lower Cretaceous (upper Albian) Cambridge Greensand. Subsequent descriptions of the fragmentary remains of ankylosaurs from the Early Cretaceous of Europe have been tentatively assigned to the genus *Polacanthus* [18].

Only nodosaurids have been described from the Upper Cretaceous of Europe with *Struthiosaurus austriacus* described from the Campanian of Austria in 1871 [19]–[24] followed by *Struthiosaurus transylvanicus* [25], [26], [27] from the uppermost Cretaceous (upper Maastrichtian) strata of Romania. Until recently, all Late Cretaceous ankylosaur fossils in Europe have been assigned to *Struthiosaurus* [28]–[30] including *Struthiosaurus languedocensis* from the Campanian of southern France [31]. The primitive nodosaurid *Hungarosaurus tormai* [32], [33] from the mid-Late Cretaceous (Santonian) is now known from multiple specimens and has become the best documented ankylosaur in Europe.

Fragmentary ankylosaur remains are also known from a number of localities from the Middle to Upper Jurassic strata of Europe, but have been relatively uninformative as specimens are based largely on isolated skeletal elements [34].

Northeastern Spain has contributed many dinosaur discoveries from both Lower and Upper Cretaceous strata in recent years [35]. The Early Cretaceous dinosaurs discovered to date include numerous sauropods, iguanodonts, and ankylosaurs from the Barremian-lower Aptian, with all the fragmentary ankylosaur material assigned tentatively to the genus *Polacanthus* [25], [28], [36]–[40]. All the Late Cretaceous ankylosaurs from Spain have in turn been assigned to *Struthiosaurus* [28]–[30].

**Figure 1:**
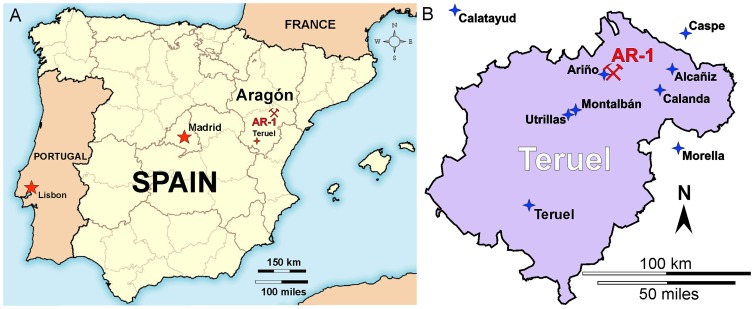
Locality maps. (A). Iberian Peninsula showing location of Santa María Coal Mine near Ariño, Teruel Province, Aragón, Spain with new dinosaur locality AR-1.(B) Teruel Province, Aragón, showing location of AR-1 east of Ariño.

The earliest reported dinosaur remains from Spain were found in the Escucha Formation, few significant vertebrate fossils had been recovered from these rocks in the 140 intervening years [41], [42]. Current research on vertebrate sites in the Escucha Formation in the northern Teruel Province in the Community of Aragón, Spain, by the Fundación Conjunto Paleontológico of Teruel-Dinópolis has resulted in the discovery of an extensive new dinosaur locality in the open-pit Santa María coal mine near Ariño (Fig. 1) operated by Sociedad Anónima Minera Catalano-Aragonesa (SAMCA Group) [42]. The most abundant dinosaur identified is a distinctive iguanodontian ornithopod recently described as *Proa valdearinnoensis* [43]. Among the many other significant fossils excavated are two associated partial skeletons of a new species of ankylosaur, described herein as *Europelta carbonensis* n. gen., n. sp. This new taxon is the most completely known ankylosaur in Europe and adds considerable new information about Early Cretaceous ankylosaurian phylogeny and biogeography.

### Geological Setting

**Figure 2:**
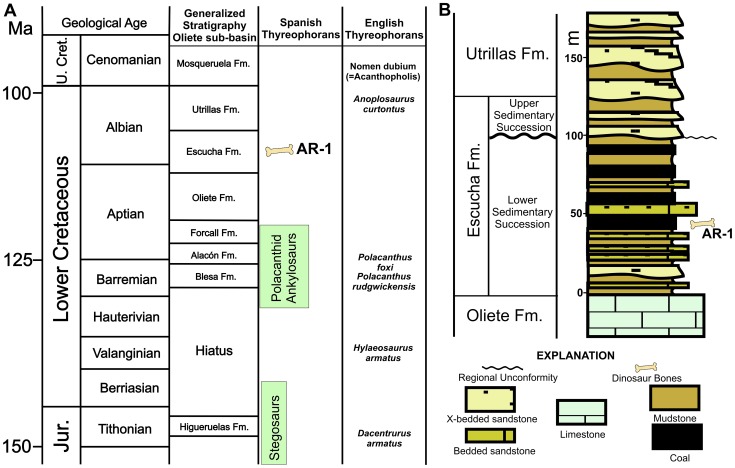
Generalized stratigraphy. (A) General Lower Cretaceous stratigraphy in the area of the Oliete sub-basin with the relative distribution of thyreophoran dinosaurs in Spain and southern England indicated. (B) General stratigraphy of the Escucha Fm. in the area around Ariño, Aragón, Spain showing approximate position of dinosaur locality AR-1. Stratigraphic nomenclature following Rodríguez-López and others [53].

Counterclockwise rotation of the Iberian Plate toward the end of the Early Cretaceous resulted in the development of a series of syndepositional sub-basins bounded by active faults within Ebro Basin south of the Pyrenean ranges, northeast of the Iberian Range, and northwest of the Catalan/Coastal Range [44], [45]. The new dinosaur locality is within the Oliete sub-basin on the northwest margin of the Escucha outcrop belt [42], [44]. The *Formación Lignitos de Escucha* and overlying *Formación Arenas de Utrillas* were initially described in 1971 [46]. These largely Albian-aged strata were deposited along the northwestern margin of the Tethys Sea during the fragmentation of this terrain, and overlie Aptian strata in the center of each sub-basin and unconformably overlie progressively older strata toward their margins. Initially, the Escucha Formation was divided into three members [47] and interpreted to be an unconformity-bounded lower to middle Albian depositional sequence, representing a progradational, tidally-dominated delta sequence [44], [48]–[52]. Recently, the upper “fluvial” member has been reinterpreted as an eolian depositional sequence separated from the underlying portions of the Escucha Formation by a regional unconformity [53]. We recognize this bipartite division of the Escucha Formation (Fig. 2).

The geologic age of the Escucha Formation has been considered to be early to middle Albian. It overlies Aptian strata in central basinal settings and is, in turn, overlain by the upper Albian Utrillas Formation [44]. However, both calcareous plankton (foraminifera and nanoplankton) [54] and palynomorphs [55], [56] indicate that the lower Escucha Formation is late Aptian in age. Both fresh and brackish coal-bearing strata are recognized below the regional unconformity within the Escucha [43]. However, reports on the microplankton restrict marine and marginal marine facies to the late Aptian in the lower Escucha Formation [54]–[56]. Marine ostracods have been reported from the upper Escucha Formation northeast of Teruel that confirm an Albian age for the upper portion of these strata in this area [57].

**Figure 3:**
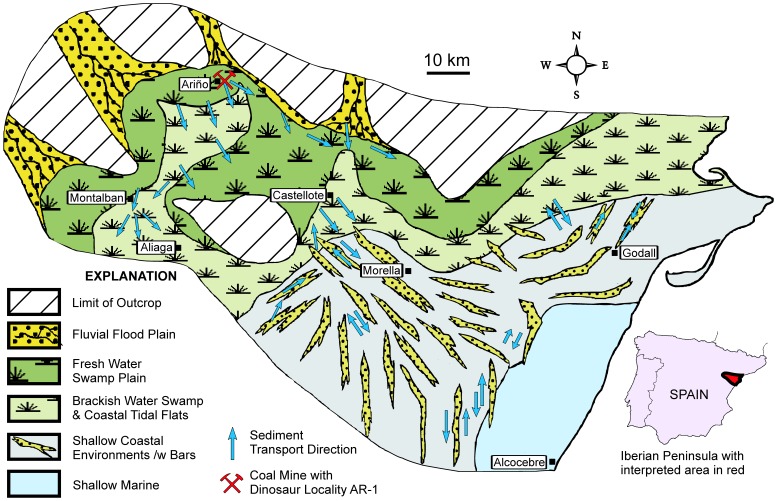
Paleogeographic reconstruction of the Escucha outcrop belt during deposition of coal under freshwater conditions in the Ariño area. Modified after Querol and others [44] with salinity data based on ostracods from Tibert and others [58].

A sample of the matrix from the bonebed was processed for both palynomorphs and calcareous microfossils. The palynomorphs were exclusively of terrestrial origin and indicated an Albian age (Gerry Waanders, 2012, personal communication). The microfossils consisted exclusively of freshwater ostracods and charophytes. The ostracods represent new species and the charophytes are also reported from the Albian of Tunisia [58]. No arenaceous foraminifera were identified, which, along with the absence of dinoflagelates, indicates that the bonebed formed well inland of marine and brackish water influences (Fig. 3).

**Figure 4:**
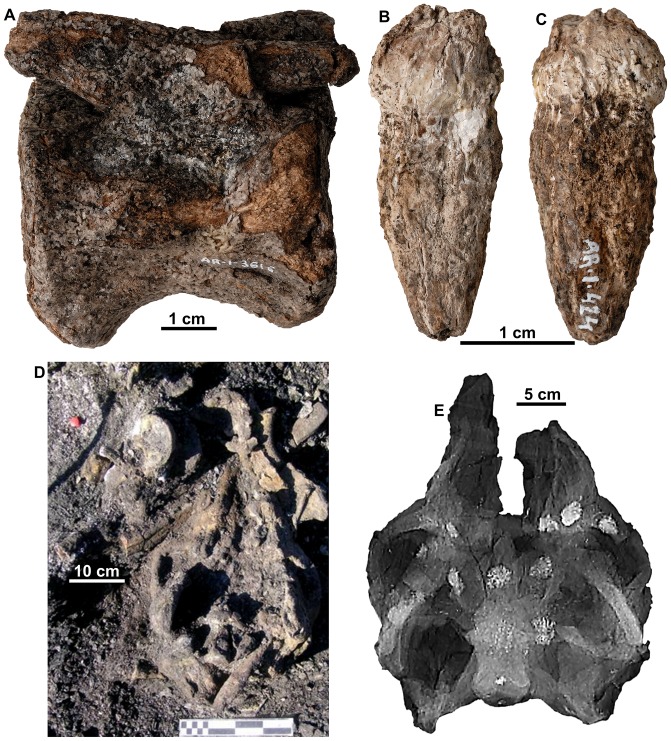
Diagenetic damage to bones on exposure to the atmosphere. Caudal vertebra AR-1-3616/31 in (A) lateral view showing damage to the bone from the growth of gypsum crystals. Maxillary tooth AR-1-424/10 in (B) labial view and (C) lingual views showing diagenetic damage to teeth. Skull of type of *Europelta carbonensis* n. gen., n. sp., AR-1-544/10 (D) as exposed in AR-1/10 and (E) X-ray image in dorso-ventral orientation. Light patches are areas of pyrite mineralization.

The bonebed is located immediately below the lowest mineable coal seam in the Santa María coal mine (Fig. 2), in a dark olive-gray to olive-black mudstone that preserves a high percentage of fossil plant debris. In overall appearance, the rock is much like the plant debris beds in the Wessex Formation on the Isle of Wight [59], [60] and, as in those beds, there is a great amount of pyrite (iron sulfide) disseminated through the matrix and in the fossils. Significant amounts of iron sulfide in the coals were found to decrease up section, away from marine and brackish-water environments. In addition to this depositional relationship, it has been speculated that detrital evaporites from exposed Triassic strata on the north and northwest sides of the basin have secondarily contributed significant amounts of sulfur to these coals [43], [61]. Additionally, the abundance of pyrite in the bones indicates that the long-term stability of the fossils is in question as pyrite breaks down in an expansive oxidation reaction that liberates corrosive sulfuric acid compounds that cannot be reversed [62]. The degradation by this pyrite is apparent on most of the bones soon after exposure to the surface. This is indicated by the rapid appearance of fine, powdery to crystalline gypsum coating bones and teeth, and by the expansion and shattering of some bones and teeth with internal gypsum formation (Fig. 4). Protocols are being developed to ensure the preservation of the primary data represented by these important fossils [42], [62].

The bonebed was located many tens of meters underground prior to strip mining operations in the Santa María coal mine. As mining operations proceed, more of the plant debris stratum containing the bonebed is exposed as simultaneous reclamation covers the previously exposed surface. Thus, with the help of mine managers, efficient methodologies for the documentation and extraction of significant fossils have been established [42]. By the end of 2012, an area of approximately 25 ha had been investigated and the areal distributions of 101 vertebrate concentrations were documented; 33 of these consisted of associated dinosaur skeletons (mostly iguanodonts) and 68 consisted of other vertebrate remains (mostly turtles and crocodilians). During this stage of the project, numerous dinosaurs (ornithischian elements and associated skeletons, and saurischian teeth), two types of turtle, crocodilians, fish (both ostheicthyians and selachiens), coprolites, molluscs (freshwater bivalves and gastropods), arthropods (ostracods), and abundant plant remains (logs, plant fragments, palynomorphs, and amber) have been excavated.

The bonebed designated AR-1 contains more than 5000 identifiable vertebrate specimens recovered from isolated skeletal remains and associated individual animals. All fossils receive a consecutive number from the site, each association is numbered as well. Thus:

- AR-1-#fossil identifies each fossil found at the Ariño site (the ID written on each fossil);
- AR-1/#concentration identifies a collection of bones belonging to a single skeleton;
- AR-1-#fossil/#concentration identifies a fossil from a bone concentration # belonging or not belonging to a single skeleton.

**Figure 5:**
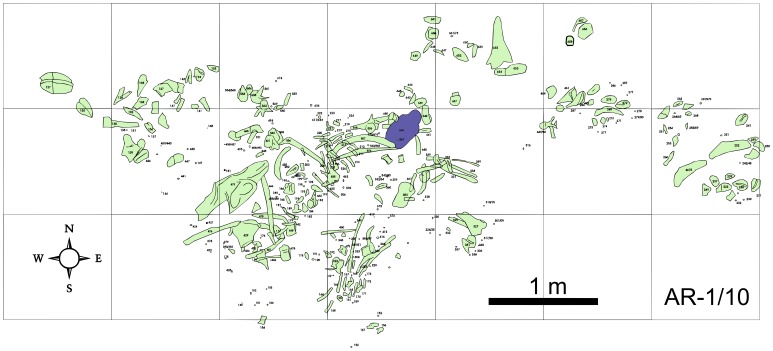
Quarry sketch map of AR-1/10. Bones and armor shaded in green and skull shaded in purple.

**Figure 6:**
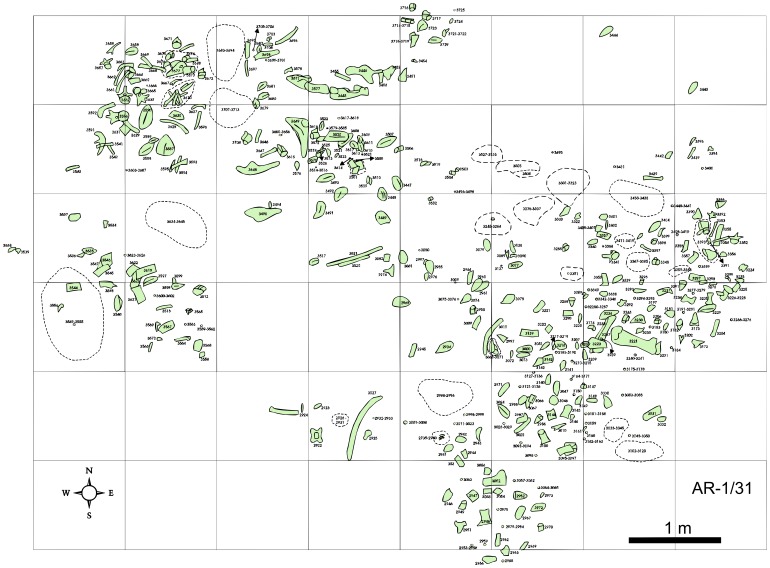
Quarry sketch map of AR-1/31. Bones and armor shaded in green.

The two associated ankylosaur skeletons described herein were separated by 200 meters. The location of the holotype AR-1/10 (Fig. 5) was still available for examination and sampling for microfossils in December of 2011 [58], while that of the paratype AR-1/31 (Fig. 6) was already inaccessible.

## Materials and Methods

### Paleontological Ethics Statement

All of the specimens described in this paper (AR-1/10 and AR-1/31) are reposited in the collections of the Fundación Conjunto Paleontológico de Teruel-Dinópolis/Museo Aragonés de Paleontología (FCPTD/MAP). Locality information is available from the registrar of the museum as per museum policy. All necessary permits were obtained for the described study, which complied with all relevant regulations. All of these specimens were collected under permits obtained from the Sociedad Anónima Minera Catalano-Aragonesa.

### Nomenclatural Acts

The electronic edition of this article conforms to the requirements of the amended International Code of Zoological Nomenclature, and hence the new names contained herein are available under that Code from the electronic edition of this article. This published work and the nomenclatural acts it contains have been registered in ZooBank, the online registration system for the ICZN. The ZooBank LSIDs (Life Science Identifiers) can be resolved and the associated information viewed through any standard web browser by appending the LSID to the prefix “http://zoobank.org/”. The LSID for this publication is: urn:lsid:zoobank.org:pub:9246FFA7-6271-4734-8E01-5590BE4A80C2. The LSID for *Europelta carbonensis* is: urn:lsid:zoobank.org:act:089040A3-1BCF-42D1-B99F-94840E2BB96D. The electronic edition of this work was published in a journal with an ISSN (1932-6203), and has been archived and is available from the following digital repositories: LOCKSS (http://www.lockss.org); PubMed Central (http://www.ncbi.nlm.nih.gov/pmc).

### Terminology

We do not refer to the “armor” on the skull roof as caputegulae, as we consider these patterns in the Nodosauridae to reflect impressions of scale boundaries on the skull roof as opposed to thickened remodeled cranial bone. We use the term caudal rib instead of caudal transverse process. We employ the monophyletic clade Polacanthidae of Carpenter [63] to facilitate comparison with and discussion of a number of similar taxa (*Gargoyleosaurus*, *Mymoorapelta*, *Hylaeosaurus*, *Polacanthus*, *Hoplitosaurus*, and *Gastonia*). The most recent analysis of polacanthids as a monophylogenetic subfamily of nodosaurids was by Yang and others [64], who similarly defined them as the most inclusive clade containing *Polacanthus foxii* but not A*nkylosaurus magniventris* or *Panoplosaurus mirus*.

### Institutional Abbreviations

**AMNH**, American Museum of Natural History, New York, New York, **NHMUK**, Natural History Museum, London, England, **CEUM**, Prehistoric Museum, Utah State University, Price, Utah, **DMNH**, Denver Museum of Nature and Science, Denver, Colorado, **MPC**, Geological Institute, Ulaan Bataar, Mongolia, **FCPTD/MAP**, Fundación Conjunto Paleontológico de Teruel-Dinópolis/Museo Aragonés de Paleontología, Teruel, Spain, **FMNH**, Field Museum of Natural History, Chicago, **MPC**, Institute of Geology, Mongolian Academy of Sciences, Ulaan Baatar, Mongolia; **INBR**, Victor Valley Museum, Apple Valley, California, **IVPP**, Institute of Vertebrate Paleontology and Paleoanthropology, Beijing, China, **KUVP**, Kansas Museum of Natural History, Lawrence, Kansas, **MPC**, Mongolian Paleontological Center, Ulaan Baatar, Mongolia; **MNA**, Museum of Northern Arizona, Flagstaff, Arizona, **NMC**, National Museum of Canada, Ottawa, Canada, **NMW**, National Museum of Wales, Cardiff, England, **PIN**, National Institute of Paleontology, Moscow, Russia, **QM**, Queensland Museum, Queensland, Australia, **ROM**, Royal Ontario Museum, Toronto, Canada, **SDNHM**, San Diego Natural History Museum, San Diego, California, **SGDS**, Saint George Dinosaur Discovery Site at Johnson Farm, St. George, Utah, **SMP**, State Museum of Pennsylvania, Harrisburg, Pennsylvania, **SMU**, Schuler Museum, Southern Methodist University, Dallas, Texas, **USNM**, National Museum of Natural History, Smithsonian Institution, Washington D.C.

### Comparative Material

In addition to accessing the ever-expanding ankylosaur literature, the senior and third authors have had the opportunity to study firsthand much of the important ankylosaur material collected globally. From the basal thyreophorans: the type material of *Scutellosaurus lawleri* (MNA P1.175), the type material of *Scelidosaurus harrisoni* (NHMUK R 1111), and a large, exceptionally well-preserved, articulated *Scelidosaurus* specimen with intact armor, collected and owned by David Sole and currently exhibited at the University of Bristol. Also, a full cast of the left side of the skeleton (SGDS 1311) exhibited in southwestern Utah was examined.

In regards to Jurassic ankylosaurs: the extensive type and paratype material of *Mymoorapelta maysi* housed at the Museum of Western Colorado, *Gargoyleosaurus parkpinorum* (DMNH 27726), and the dentary of *Sarcolestes leedsi* (NHMUK R 2682) were studied.

Early Cretaceous polacanthine ankylosaur material examined includes *Polacanthus foxii* (NHMUK R 175, 9293), *Hylaeosaurus armatus* (NHMUK R 3775), *Hoplitosaurus marshi* (USNM 4752), and the extensive material of *Gastonia burgei* material housed at the Prehistoric Museum (including holotype CEUM 1307 and paratype material), and cranial material from a minimum of six individuals at Brigham Young University's Earth Science Museum, together with the postcranial skeleton of an unnamed new species of polacanthine (BYU 245).

Among basal shamosaurine-grade ankylosaurids, *Cedarpelta bilbyhallorum* (including CEUM 12360 and paratype material), *Shamosaurus scutatus* (PIN 3779/2), and a cast of the skull of *Gobisaurus domoculus* (IVPP 12563) housed at the Royal Tyrell Museum were studied.

Among derived North American ankylosaurs, *Nodocephalosaurus kirtlandensis* (SMP-VP-900), *Ankylosaurus magniventri*s (AMNH 5214, 5859; NMC 8880), *Anadontosaurus lambei* (NMC 8530), *Dyoplosaurus acutosquameus* (ROM 784), *Scolosaurus cutleri* (NHMUK, R 5161), and several important examples of *Euoplocephalus tutus*, (AMNH 5404, 5409; RTMP 91.127.1) were examined.

Asian ankylosaur material researched include an adult skull of *Tsagantegia longicranialis* (MPC 100/1306), China, *Pinacosaurus granger*i (AMNH 6523) and three undescribed skulls personally excavated by JIK from the Djadokhta Formation, Shabarakh Usu (Flaming Cliffs, Mongolia) and housed at MAS, *Talarurus plicatospineus* (composite skeleton made up of parts of many individuals assigned to PIN 557), cast skull of *Saichania chulsanensis* (PIN 3141/251), a relatively complete specimen referred to *Saichania* with in situ armor but lacking its skull (MPC 100/1305), *Tarchia gigantea* (PIN 3142/250), a cast skull of *Minotaurasaurus ramachandrani* (INBR 21004), and a cast skeleton of *Crichtonsaurus benxiensis* housed in the Museum at the Chaoyang Bird National Geopark, Liaoning.

Numerous nodososaurids were examined, including the Early Cretaceous nodosaurids *Sauropelta edwardsi* (AMNH, 3016, 3032, 3035, 3036; YPM 5502, 5529, 5499, 5178), *Peloroplites cedrimontanus* (CEUM 26331 and the extensive paratype material), and *Pawpawsaurus campbelli* (SMU 73203; = “*Texasestes*” *pleurohalio* USNM 337987). The early Late Cretaceous nodosaurids reviewed include *Animantarx ramaljonesi* (CEUM 6228), *Silvisaurus condrayi* (KUVP 10296), *Nodosaurus textilis* (YPM 1815), and *Stegopelta landerensis*(FMNH UR88) and the Late Cretaceous nodosaurids *Panoplosaurus mirus* (NMC 2759), *Edmontonia rugosidens* (USNM 11868; AMNH 5665), *Edmontonia longiceps* (NMC 8531), *Denversaurus schlessmani* (DMNH 468), casts of *Struthiosaurus austriacus* at the Carnegie Museum (PIUW 2349) and *Struthiosaurus transylvanicus* (NHMUK R 4966).

Enigmatic taxa such as the skull of *Minmi paravertebrata* (QM F18101), the skeleton of *Liaoningosaurus paradoxus* (IVPP V12560), and *Aletopelta coombsi* (SDNHM 33909) were also examined.

## Results

### Systematic Paleontology

Dinosauria Owen, 1842 [65]

Ornithischia Seeley, 1887 [66]

Thyreophora Nopcsa, 1915 [25]

Ankylosauria Osborn, 1908 [67]

Nodosauridae Marsh, 1890 [68]

Struthiosaurinae Nopcsa, 1923 [69]

### Diagnosis

Nodosaurid ankylosaurs that share a combination of characters including: narrow predentaries; a nearly horizontal, unfused quadrates that are oriented less than 30° from the skull roof, and condyles that are 3 times transversely wider than long; premaxillary teeth and dentary teeth that are near the predentary symphysis; dorsally arched sacra; an acromion process dorsal to midpoint of the scapula-coracoid suture; straight ischia, with a straight dorsal margin; relatively long slender limbs; a sacral shield of armor; and erect sacral armor with flat bases. **Struthiosaurinae is defined as the most inclusive clade containing** ***Europelta*** **but not** ***Cedarpelta*** **,** ***Peloroplites, Sauropelta*** **or** ***Edmontonia*** **.**

***Europelta*** Kirkland, Alcalá, Loewen, Espílez, Mampel, and Wiersma 2013 **gen. nov.**

urn:lsid:zoobank.org:act:62808E3D-85BE-4AE3-B771-9CFF2C6AC054

### Etymology

*“Euro”* as a contraction for Europe in regard to its origin and “*pelta*” Greek for shield, a common root for ankylosaurian genera; “Europe's shield”.

### Diagnosis

Same as for the only known species below.

***Europelta carbonensis*** Kirkland, Alcalá, Loewen, Espílez, Mampel, and Wiersma 2013 **gen. et sp. nov.**

urn:lsid:zoobank.org:act:089040A3-1BCF-42D1-B99F-94840E2BB96D

Figures 7-33

**Figure 7:**
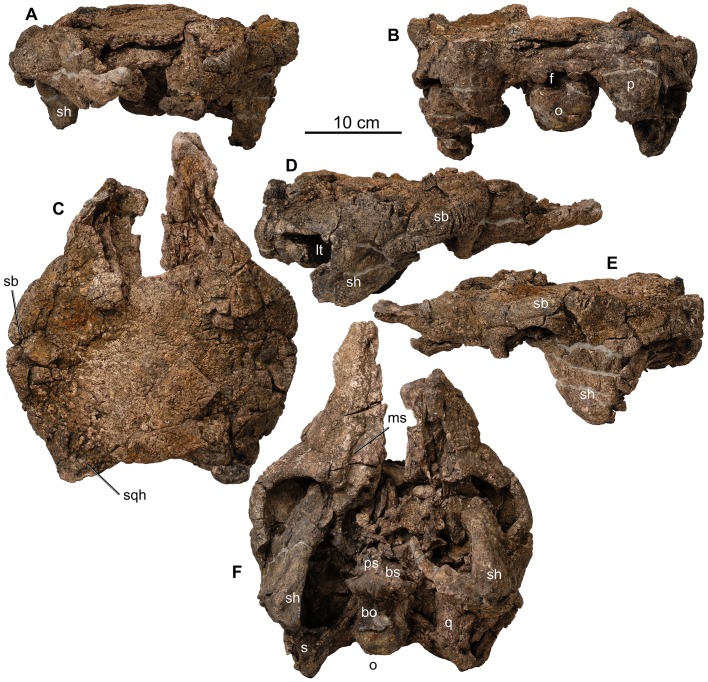
Skull of type of *Europelta carbonensis* n. gen., n. sp., AR-1-544/10. Partial skull in: (A) anterior view, (B) posterior view, (C) dorsal view, (D) right lateral view, (E) left lateral view, and (F) ventral view. Abbreviations: bo = basioccipital, bs = basisphenoid, f = foramen magnum, lt = lower temporal fenestra, o = occipital condyle, p = paraocciptal process, ps = parasphenoid, q = quadrate, s = squamosal, sb = supraorbital boss, sh = suborbital horn, sqh = squamosal horn.

**Figure 8:**
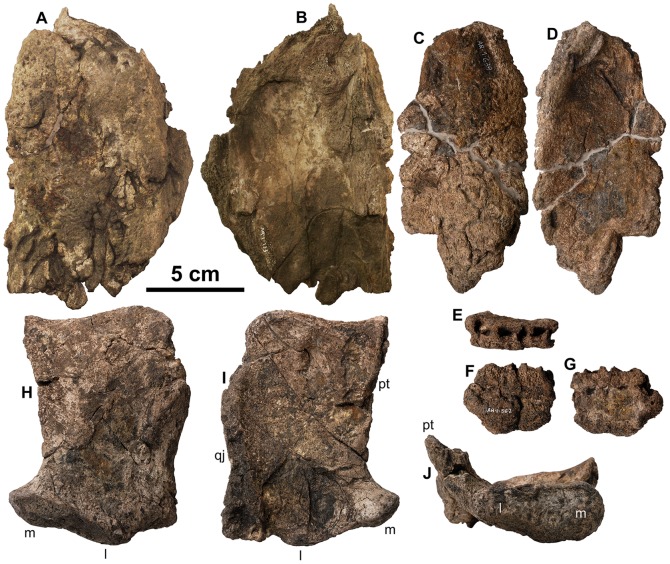
Isolated skull fragments from type of *Europelta carbonensis* n. gen., n. sp., AR-1-544/10. Right nasal AR-1/10 in: (A) dorsal view and (B) ventral view. Left nasal AR-1-639/10 in: (C) dorsal and (D) ventral view. Dentary fragment in AR-1-362/10 in: (E) dorsal view, (F) medial view, and (G) lateral view. Isolated right quadrate AR-1-544*/10 in: (H) posterior view, (I) anterior view, and (J) ventral view. Abbreviations: l = lateral condyle, m = medial condyle, qj = suture for quadratojugal, pt = broken margin of pterygoid.

**Figure 9:**
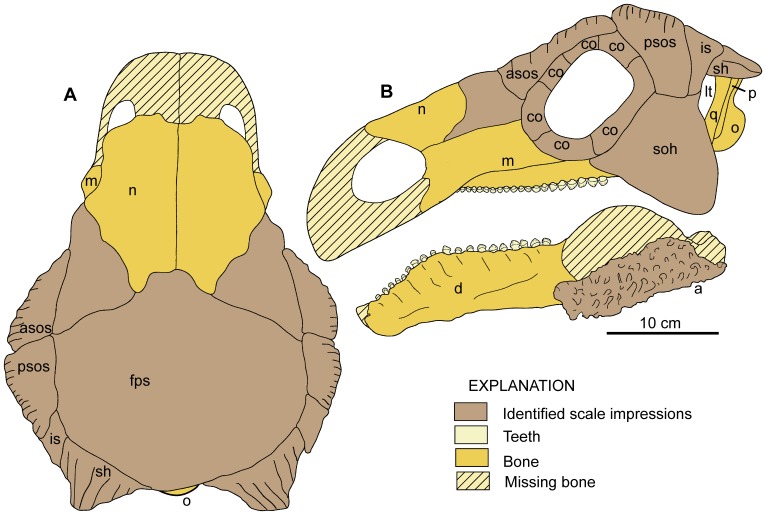
Drawing of *Europelta carbonensis* n. gen., n. sp., skull reconstruction. *Europelta* skull reconstruction in: (A) dorsal view and (B) left lateral view with reconstruction of mandible. Abbreviations: a = angular, asos = anterior supraorbital scale, co = circumorbital scales, d = dentary, fps = frontoparietal scale, is = intermediate scale, lt = lower temporal fenestra, m = maxilla, n = nasal, o = occipital condyle, p = paraocciptal process, psos = posterior supraorbital scale, sh = squamosal horn, soh = suborbital horn.

**Figure 10:**
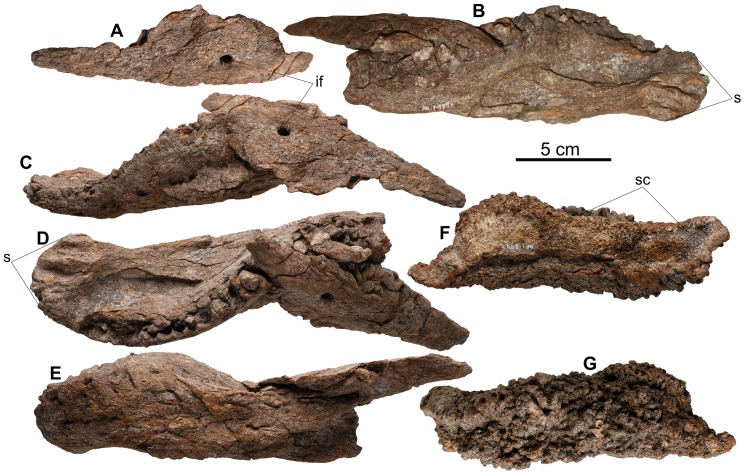
*Europelta carbonensis* n. gen., n. sp. mandible from AR-1/31. Fused dentary and splenial AR-1-3698/31: Isolated splenial in (A) medial view. Dentary in: (B) medial view, (C) dorsal view with splenial inverted in medial view, and (D) dorso-medial view with splenial inverted in medio-ventral view, and (E) latero-ventral view with posterior splenial visible in dorsal view. Angular AR-1-2945/31 in: (F) lateral view and (G) medial view. Abbreviations: if = intermandibular foramen. s = mandibular symphysis, sc = splenial contact.

**Figure 11:**
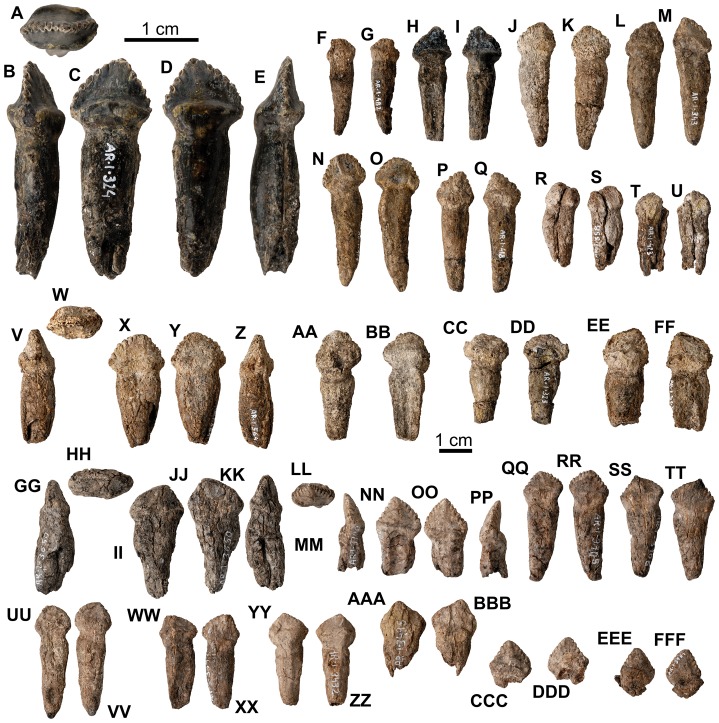
*Europelta carbonensis* n. gen., n. sp. teeth. Maxillary teeth from holotype of *Europelta carbonensis* n. gen., n. sp., AR-1/10 (A-FF). Well-preserved tooth AR-1-324/10 in: (A) occlusal view, (B) distal view, (C) lateral view, (D) ligual view, and (E) anterior view. AR-1-482/10 in: (F) labial view and (G) ligual view. AR-1-325/10 in: (H) labial view and (I) ligual view. AR-1-563/10 in: (J) labial view and (K) ligual view. Possible premaxillary tooth AR-1-343/10 in: (L) labial view and (M) ligual view. AR-1-417/10 in: (N) labial view and (O) ligual view. AR-1-418/10 in: (P) labial view and (Q) ligual view. AR-1-358/10 in: (R) labial view and (S) ligual view. AR-1-423/10 in: (T) labial view and (U) ligual view. AR-1-564/10 in: (V) posterior view, (W) occlusal view, (X) labial view, (Y) ligual view, and (Z) mesial view. AR-1-428/10 in: (AA) labial view and (BB) ligual view. AR-1-323/10 in: (CC) labial view and (DD) ligual view. AR-1-567/10 in: (EE) labial view and (FF) ligual view. Dentary teeth from type of *Europelta carbonensis* n. gen., n. sp., AR-1/31 (GG-FFF). AR-1-3650/31 in: (GG) posterior view, (HH) occlusal view, (II) labial view, (JJ) ligual view, and (KK) mesial view. AR-1-3700/31 in: (LL) occlusal view, (MM) posterior view, (NN) labial view, (OO) ligual view, and (PP) mesial view. AR-1-3705/31 in: (QQ) labial view and (RR) ligual view. AR-1-3706/31 in: (SS) labial view and (TT) ligual view. AR-1-3524/31 in: (UU) labial view and (VV) ligual view. AR-1-3699/31 in: (WW) labial view and (XX) ligual view. AR-1-3432/31 in: (YY) labial view and (ZZ) ligual view. AR-1-3495/31 in: (AAA) labial view and (BBB) ligual view. AR-1-3701/31 in: (CCC) labial view and (DDD) ligual view. AR-1-3961/31 in: (EEE) labial view and (FFF) ligual view.

**Figure 12:**
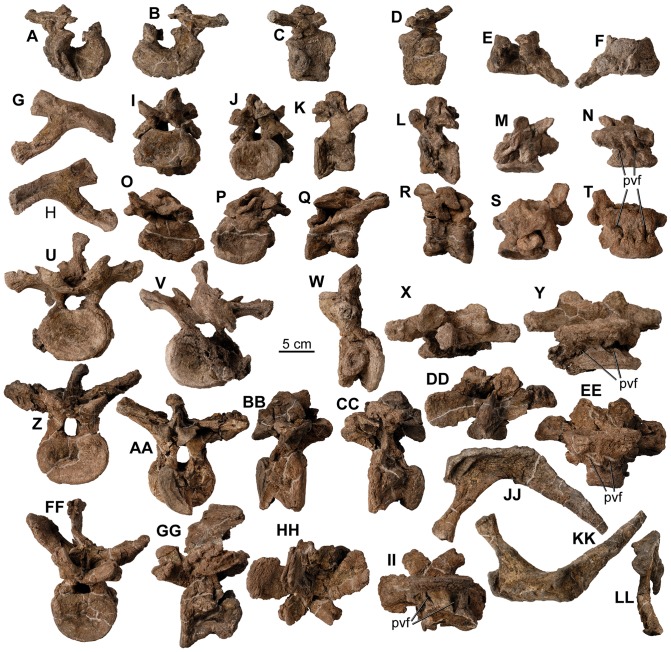
Cervical vertebrae and ribs of holotype of *Europelta carbonensis* n. gen., n. sp. AR-1/10. Atlas AR-1-649/10 in: (A) anterior view, (B) posterior view, (C) right lateral view, (D) left lateral view, (E) dorsal view, and (F) ventral view. Mid-cervical rib AR-1-450/10 in: (G) anterior view and (H) posterior view. Anterior cervical vertebra AR-1-650/10 in: (I) anterior view, (J) posterior view, (K) right lateral view, (L) left lateral view, (M) dorsal view, and (N) ventral view. Anterior cervical vertebra AR-1-637/10 in: (O) anterior view, (P) posterior view, (Q) right lateral view, (R) left lateral view, (S) dorsal view, and (T) ventral view. Mid-cervical vertebra AR-1-449/10 in: (U) anterior view, (V) posterior view, (W) right lateral view, (X) dorsal view, and (Y) ventral view. Mid-cervical vertebra AR-1-431/10 in: (Z) anterior view, (AA) posterior view, (BB) left lateral view, (CC) right lateral view, (DD) dorsal view, and (EE) ventral view. Posterior cervical vertebra AR-1-533/10 in: (FF) anterior view, (GG) left lateral view, (HH) dorsal view, and (II) ventral view. Posterior right cervical rib AR-1-4452/10 in: (JJ) posterior view, (KK) anterior view, and (LL) ventral view. Abbreviation pvf = paired ventral fossae.

**Figure 13:**
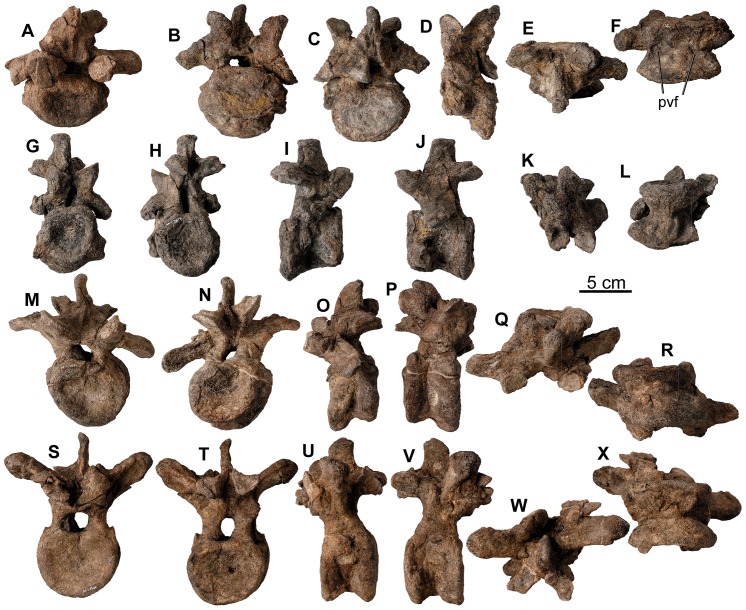
Cervical vertebrae of *Europelta carbonensis* n. gen., n. sp AR-1/31. Anterior cervical vertebra AR-1-3657/31 in: (A) anterior view. Anterior cervical vertebra AR-1-3671/31 in: (B) anterior view, (C) posterior view, (D) left lateral view, (E) dorsal view, and (F) ventral view. Mid-cervical vertebra AR-1-3676/31 in: (G) anterior view, (H) posterior view, (I) right lateral view, (J) left lateral view, (K) dorsal view, and (L) ventral view. Posterior cervical vertebra AR-1-3632/31 in: (M) anterior view, (N) posterior view, (O) right lateral view, (P) left lateral view, (Q) dorsal view, and (R) ventral view. Posterior cervical vertebra AR-1-3586/31 in: (S) anterior view, (T) posterior view, (U) right lateral view, (V) left lateral view, (W) dorsal view, and (X) ventral view. Abbreviation pvf = paired ventral fossae.

**Figure 14:**
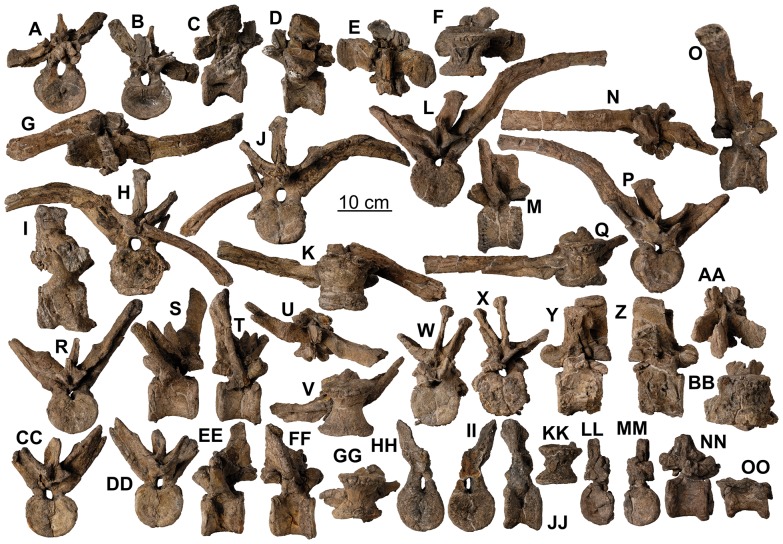
Dorsal vertebrae of holotype of *Europelta carbonensis *n. gen., n. sp. AR-1/10. Anterior dorsal vertebra AR-1-638/10 in: (A) anterior view, (B) posterior view, (C) right lateral view, (D) left lateral view, (E) dorsal view, and (F) ventral view. Anterior dorsal vertebra with fused rib fragments AR-1-535/10 in: (G) dorsal view, (H) anterior view, (I) right lateral view, (J) posterior view, and (K) ventral view. Anterior dorsal vertebra with fused rib fragment AR-1-478/10 in: (L) posterior view, (M) right lateral view, (N) dorsal view, (O) left lateral view, (P) anterior view, and (Q) ventral view. Anterior dorsal vertebra with bases of fused ribs AR-1-448/10 in: (R) posterior view, (S) left lateral view, (T) right lateral view, (U) dorsal view, and (V) ventral view. Mid-dorsal vertebra AR-1-430/10 in: (W) anterior view, (X) posterior view, (Y) left lateral view, (Z) right lateral view, (AA) dorsal view, and (BB) ventral view. Mid/dorsal vertebra with bases of fused ribs AR-1-322/10 in: (CC) anterior view, (DD) posterior view, (EE) left lateral view, (FF) right lateral view, and (GG) ventral view. Mid-dorsal vertebra AR-1-566/10 in: (HH) posterior view, (II) anterior view, (JJ) left lateral view, and (KK) ventral view. Posterior dorsal vertebra AR-1-155/10 in: (LL) anterior view, (MM) posterior view, (NN) right lateral view, and (OO) ventral view.

**Figure 15:**
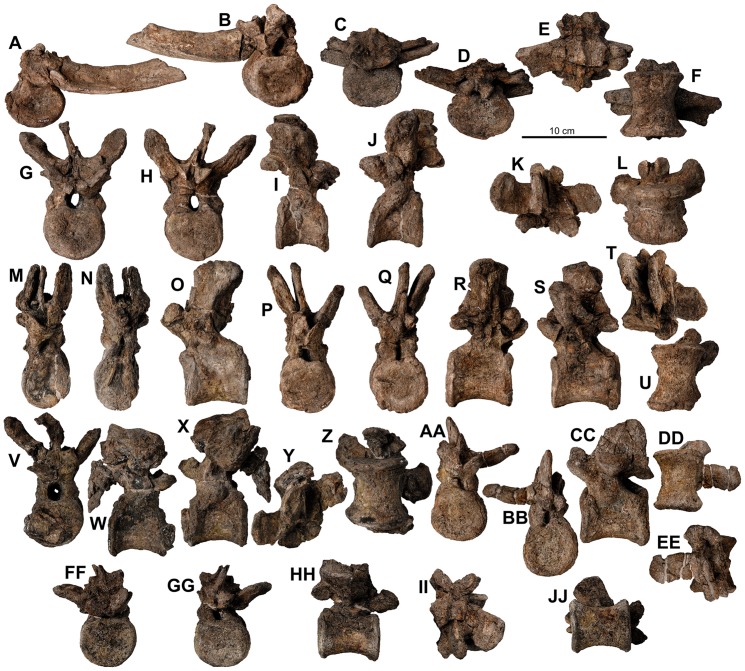
Dorsal vertebrae of *Europelta carbonensis* n. gen., n. sp. AR-1/31. Anterior dorsal vertebra with rib fragment AR-1-3662/31 in: (A) posterior view, (B) cranial view. Anterior dorsal vertebra AR-1-3672/31 in: (C) cranial view, (D) posterior view, (E) dorsal view, and (F) ventral view. Medial cervical vertebra AR-1-3633/31 in: (G) cranial view, (H) posterior view, (I) right lateral view, (J) left lateral view, (K) dorsal view, and (L) ventral view. Medial dorsal vertebra AR-1-3674/31 in: (M) cranial view, (N) posterior view, and (O) left lateral view. Medial dorsal vertebra AR-1-3489/31 in: (P) cranial view, (Q) posterior view, (R) right lateral view, (S) left lateral view, (T) dorsal view, and (U) ventral view. Medial dorsal vertebra AR-1-3675/31 in: (V) cranial view, (W) right lateral view, (X) left lateral view, (Y) dorsal view, and (Z) ventral view. Mid-dorsal vertebra AR-1-3704/31 in: (AA) cranial view, (BB) posterior view, (CC) left lateral view, (DD) ventral view, and (EE) dorsal view. Mid-dorsal vertebra AR-1-3673/31 in: (FF) cranial view, (GG) posterior view, (HH) right lateral view, (II) dorsal view, and (JJ) ventral view.

**Figure 16:**
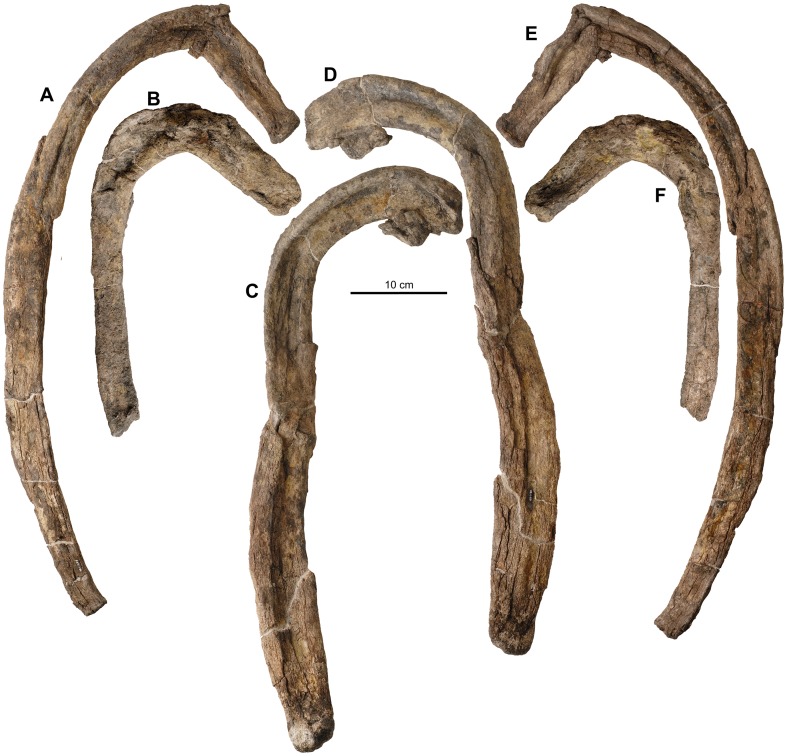
Ribs of holotype of *Europelta carbonensis* n. gen., n. sp. AR-1/10. Complete rib AR-1-476/10 in: (A) anterior view and (E) posterior view. Partial rib AR-1-333/10 in: (B) posterior view and (F) anterior view. Partial rib AR-1-331/10 in: (C) posterior view and (D) anterior view.

**Figure 17:**
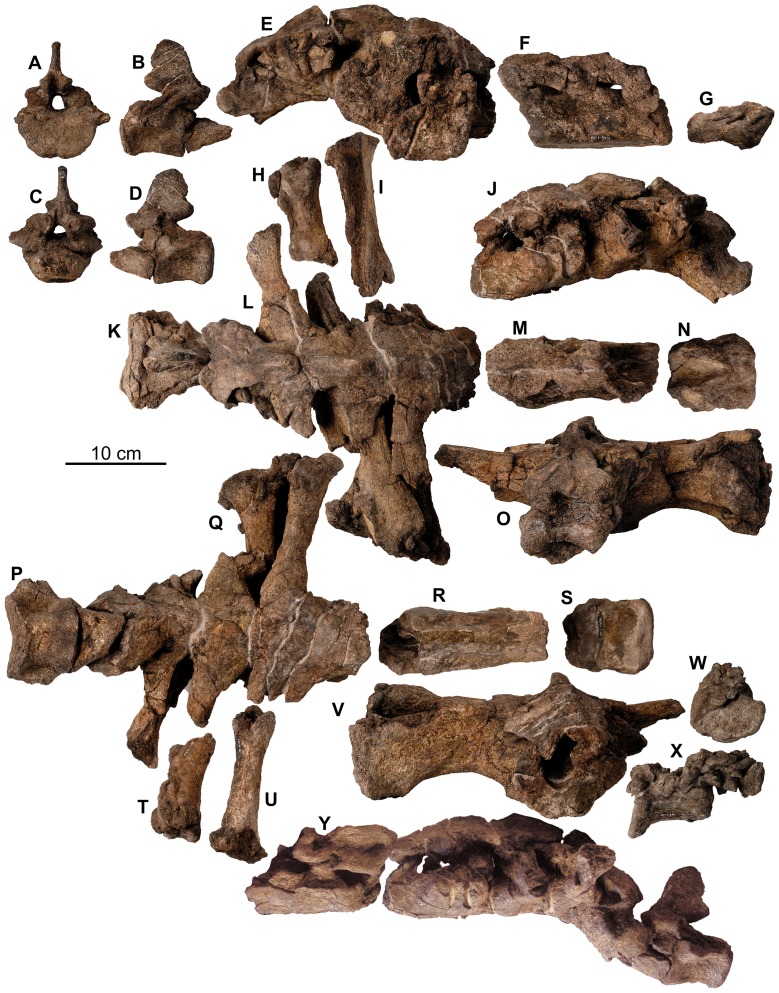
Sacrum of *Europelta carbonensis* n. gen., n. sp. AR-1/31. Caudosacral vertebra AR-1-3512/31 in: (A) posterior view, (B) right lateral view, (C) anterior view, (D) left lateral view, (K) dorsal view, and (P) ventral view. Sacrum AR-1-3446/31 in: (E) right lateral view, (J) left lateral view, (L) dorsal view, (O) anterior view, (Q) ventral view, and (V) posterior view. Medial section of synsacral rod (AR-1-3450/31) in: (F) left lateral view, (M) dorsal view, and (R) ventral view. Anteriormost centrum of synsacral rod (AR-1-3451/31) in: (G) right lateral view, (N) dorsal view, and (S) ventral view. Intermediate left sacral rib (AR-1-3460/31) in (H) posterior view and (T) posterior view. Anterior left sacral rib (AR-1-3452/31) in: (I) dorsal and (U) ventral view. Anterior end of synsacrum from AR-1/10; AR-1-154/10 in: (W) anterior view and (X) right lateral view. Initial reconstruction of the sacrum AR-1-3446, 3450, 3512/31 inverted for consistency in (Y) right lateral view.

**Figure 18:**
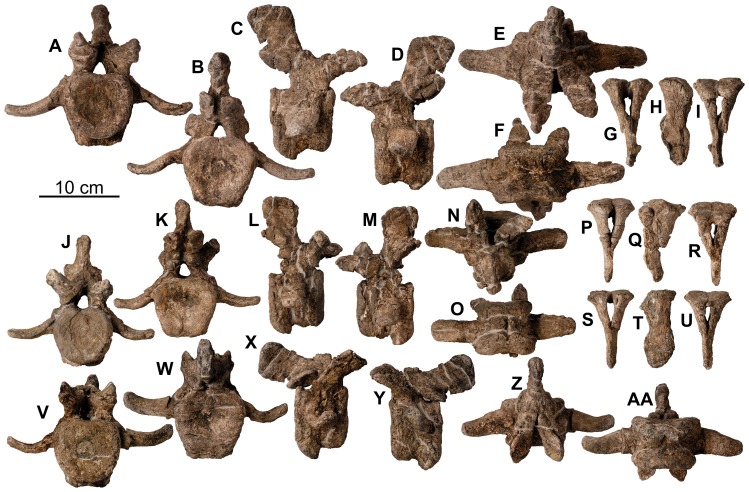
Proximal posterior vertebrae of holotype of *Europelta carbonensis* n. gen., n. sp. AR-1/10. Proximal posterior vertebra (2 or 3) AR-1-635/10 in: (A) anterior view, (B) posterior view, (C) right lateral view, (D) left lateral view, (E) dorsal view, and (F) ventral view. Proximal chevron AR-1-4451/10 in: (G) anterior view, (H) lateral view, and (I) posterior view. Proximal posterior vertebra (4 or 5) AR1-1-562/10 in: (J) anterior view, (K) posterior view, (L) right lateral view, (M) left lateral view, (N) dorsal view, and (O) ventral view. Proximal chevron AR-1-569/10 in: (P) anterior view, (Q) lateral view, and (R) posterior view. Proximal chevron AR-1-10/560 in: (S) anterior view, (T) lateral view, and (U) posterior view. Proximal posterior vertebra (5 or 6) AR-1-636/10 in: (V) anterior view, (W) posterior view, (X) right lateral view, (Y) left lateral view, (Z) dorsal view, and (AA) ventral view.

**Figure 19:**
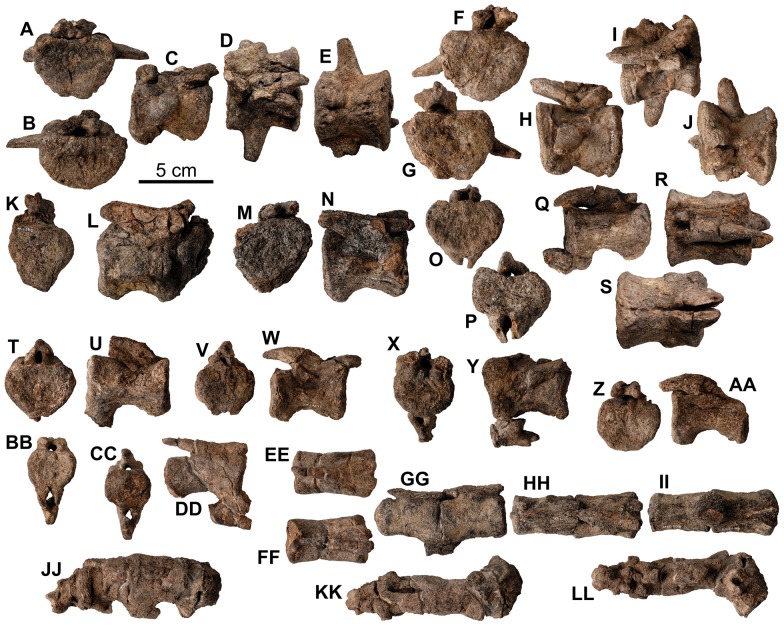
Mid- to distal caudal vertebrae of Europelta *carbonensis* n. gen., n. sp. AR-1/31. Mid-caudal vertebra AR-1-3717/31 in: (A) anterior view, (B) posterior view, (C) right lateral view, (D) dorsal view, and (E) ventral view. Mid-caudal vertebra AR-1-3348/31 in: (F) anterior view, (G) posterior view, (H) right lateral view, (I) dorsal view, and (J) ventral view. Medial posterior vertebra AR-1-3716/31 in: (K) posterior view and (L) right lateral view. Mid-caudal vertebra AR-1-3616/31 in: (M) anterior view and (N) right lateral view. Distal posterior vertebra with fused chevron AR-1-3615/31 in: (O) anterior view, (P) posterior view, (Q) right lateral view, (R) dorsal view, and (S) ventral view. Distal posterior vertebra AR-1-3478/31 in: (T) anterior view and (U) right lateral view. Distal posterior vertebra AR-1-3243/31 in: (V) anterior view and (W) right lateral view. Distal posterior vertebra with fused chevron AR-1-3206/31 in: (X) anterior view and (Y) right lateral view. Distal posterior vertebra AR-1-3265/31 in: (Z) posterior view and (AA) right lateral view. Distal posterior vertebra with fused chevron AR-1-2950/31 in: (BB) anterior view, (CC) posterior view, (DD) left lateral view, (EE) dorsal view, and (FF) ventral view. Fused pair of extreme distalmost caudal vertebrae with fused chevron AR-1-3714/31 in: (GG) right lateral view, (HH) dorsal view, and (II) ventral view. Terminal four fused posterior vertebrae with fused chevrons AR-1-3204/31 in: (JJ) right lateral view, (KK) dorsal view (KK), and (LL) ventral view.

**Figure 20:**
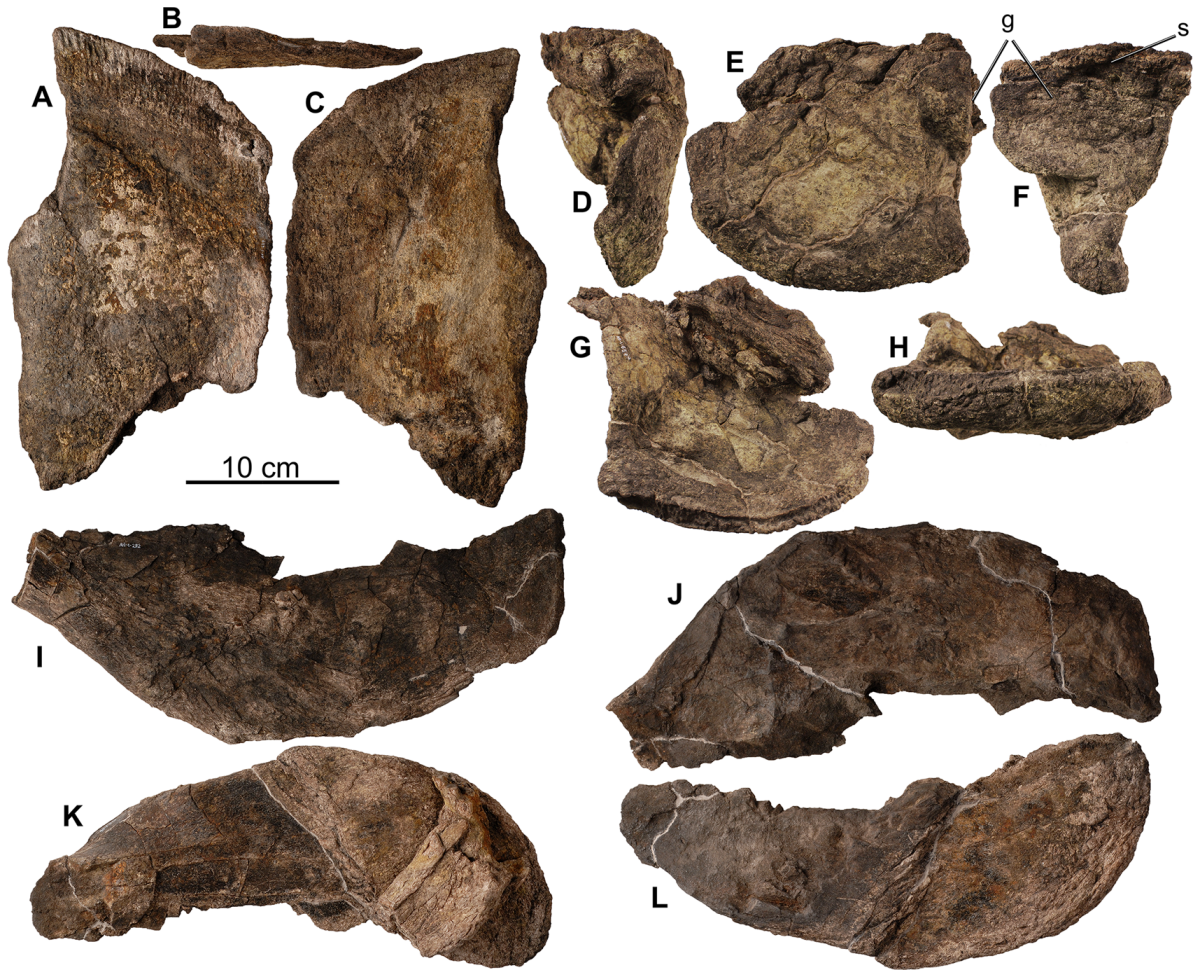
Pectoral girdle of holotype of *Europelta carbonensis* n. gen., n. sp. AR-1/10. Posterior right scapular blade AR-1-429/10 in: (A) lateral view, (B) dorsal view, and (C) medial view. Left corocoid AR-1-657/10 in: (D) anterior view, (E) lateral view, (F) posterior view, (G) medial view, and (H) ventral view. Right xiphisternal AR-1-252/10 in: (I) ventral view and (J) medial view. Left xiphisternal AR-1-4675/10 in: (K) ventral view and (L) medial view. Abbreviations: g = glenoid, s = sutural contact between corocoid and fragment of scapula.

**Figure 21:**
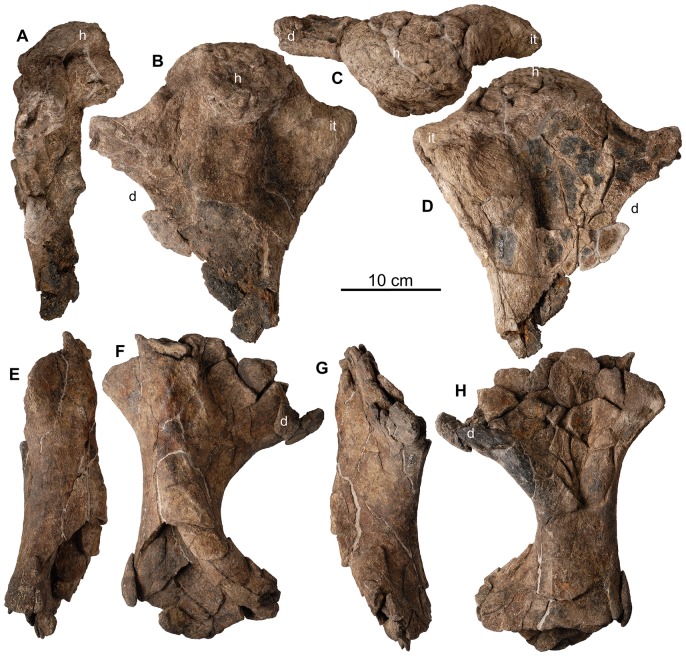
Forelimb of holotype of *Europelta carbonensis* n. gen., n. sp. AR-1/10. Left proximal humerus AR-1-655/10 in: (A) lateral view, (B) posterior view, (C) proximal view, and (D) anterior view. Shaft of right humerus AR-1-327/10 in: (E) medial view, (F) posterior view, (G) lateral view, and (H) anterior view. Abbreviations: d = deltopectoral crest, h = humeral head, it = internal tuberosity.

**Figure 22:**
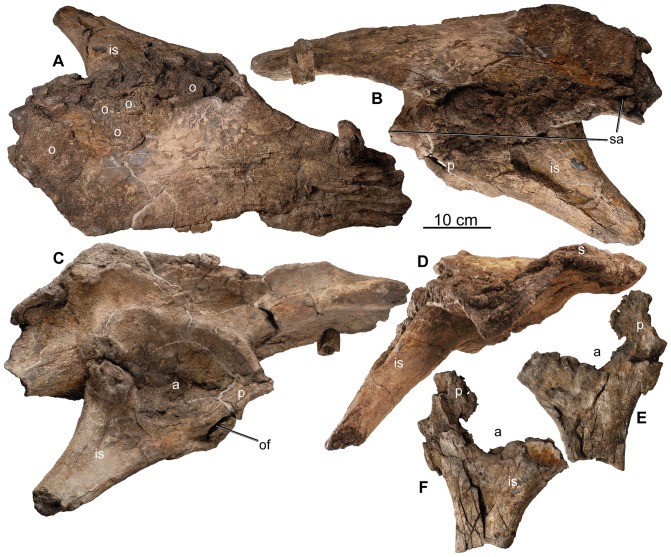
Pelvis of holotype of *Europelta carbonensis* n. gen., n. sp. AR-1/10. Right ilium with fused pubis and ischium AR-1-479/10 in: (A) dorsal view, (B) medial view, (C) ventral view, and (D) posterior view. Left ischium and fused pubis AR-1-129/10 in: (E) medial view and (F) lateral view. Abbreviations: a = acetabulum, is = ischium, of = obturator foramen between fused ischium and pubis, p = pubis, o = osteoderms, sa = sacral attachment area.

**Figure 23:**
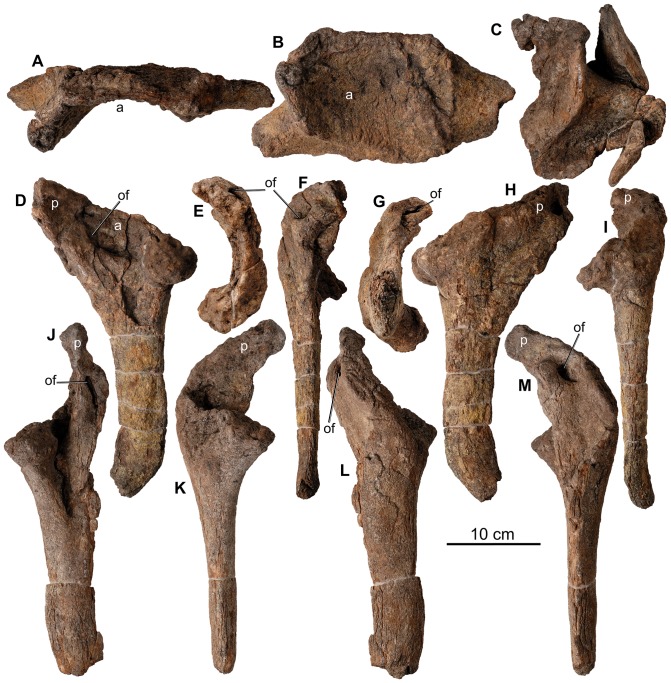
Pelvis of *Europelta carbonensis* n. gen., n. sp. AR-1/31. Fragment of ilium AR-1-3490/31 in: (A) lateral view and (B) ventral view. Fragment of ilium AR-1-3571/31 in (C) ventral view. Left ischium and fused pubis AR-1-3649/31 in: (D) lateral view, (E) proximal view, (F) anterior view, (G) distal view, (H) medial view, and (I) posterior view. Right ischium and fused pubis AR-1-3648/31 in: (J) lateral view, (K) anterior view, (L) medial view, and (M) posterior view. Red arrows indicate obturator foramen between fused ischium and pubis. Abbreviations: a = acetabulum, of = obturator foramen between fused ischium and pubis, p = pubis.

**Figure 24:**
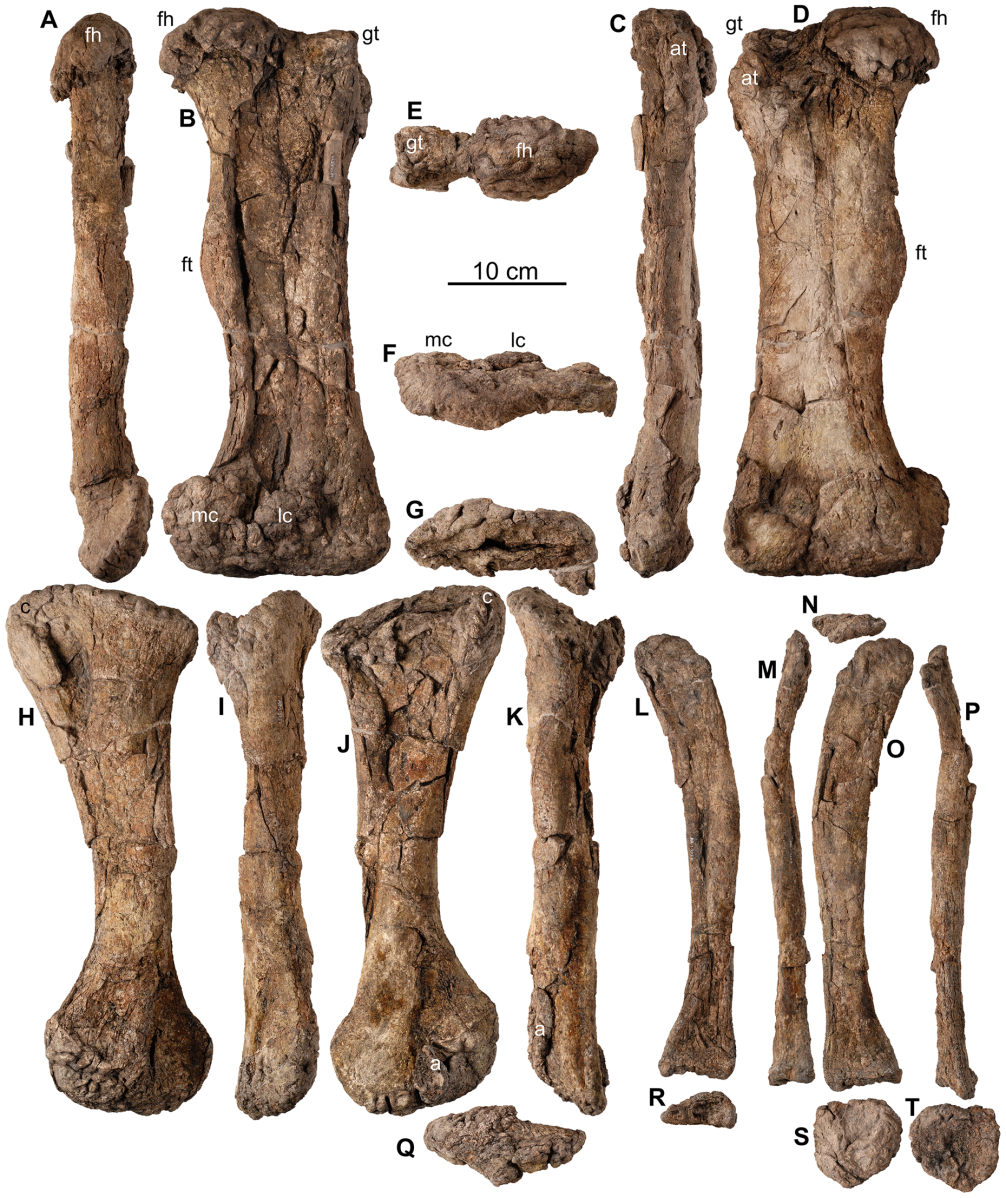
Hindlimb of *Europelta carbonensis* n. gen., n. sp. AR-1/31. Right femur AR-1-3244/31 in: (A) medial view, (B) posterior view, (C) lateral view, (D) anterior view, (E) proximal view, and (F) distal view. Right tibia AR-1-3237/31 in: (G) proximal view, (H) medial view (I), posterior view, (J) lateral view, (K) anterior view, and (Q) distal view. Right fibula AR-1-3238/31 in: (L) medial view, (M) posterior view, (N) proximal view, (O) lateral view, (P) anterior view, (R) distal view. Right calcaneum AR-1-3239/31 in: (S) lateral view, and (T) medial view. Abbreviations: a = astragalus, at = anterior trochanter, c = cnemial crest, fh = femoral head, ft = fourth trochanter, gt = greater trochanter, lc = lateral condyle, mc = medial condyle.

**Figure 25:**
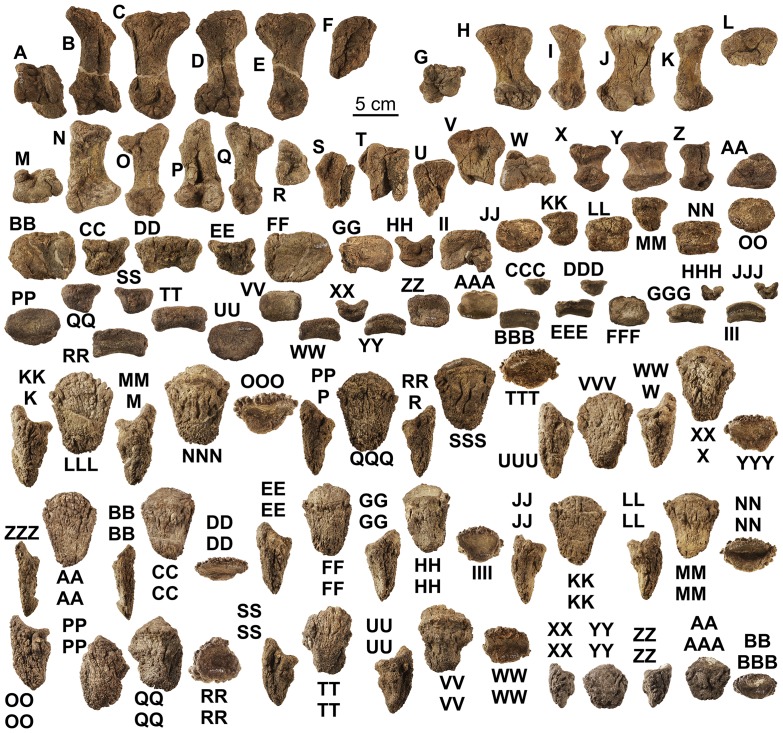
Pedal elements of *Europelta carbonensis* n. gen., n. sp. AR-1/31. Metatarsal AR-1-3100/31 in: (A) distal view, (B) anterior view, (C) right lateral view, (D) posterior view, (E) left lateral view, and (F) proximal view. Metatarsal AR-1-3234/31 in: (G) distal view, (H) anterior view, (I) right lateral view, (J) posterior view, (K) left lateral view, and (L) proximal view (L). Metatarsal AR-1-3233/31 in: (M) distal view, (N) anterior view, (O) right lateral view, (P) posterior view, (Q) left lateral view, and (R) proximal view. Possible proximal metatarsal AR-1-3173/31 in: (S) anterior view, (T) right lateral view, (U) posterior view, (V) left lateral view, and (W) anterior view. Proximal phalanx AR-1-3324/31 in: (X) right lateral view, (Y) posterior view, (Z) left lateral view, and (AA) proximal view. Medial phalanx AR-1-3174/31 in: (BB) distal view, (CC) right lateral view, (DD) posterior view, (EE) left lateral view, and (FF) proximal view. Medial phalanx AR-1-3066/31 in: (GG) distal view, (HH) right lateral view, and (II) proximal view. Medial phalanx AR-1-3032/31 in: (JJ) distal view, (KK) anterior view, (LL) right lateral view, (MM) posterior view, (NN) left lateral view, and (OO) proximal view. Distal phalanx AR-1-3292/31 in: (PP) distal view, (QQ) anterior view, (RR) right lateral view, (SS) posterior view, (TT) left lateral view, and (UU) proximal view. Distal phalanx AR-1-3356/31 in: (VV) distal view, (WW) anterior view, (XX) right lateral view, (YY) posterior view, and (ZZ) proximal view. Distal phalanx AR-10-3179/31 in: (AAA) distal view, (BBB) anterior view, (CCC) right lateral view, (DDD) posterior view, (EEE) left lateral view, and (FFF) proximal view. Distal phalanx AR-1-3224/31 in: (GGG) anterior view, (HHH) right lateral view, (III) posterior view, and (JJJ) left lateral view. Pedal ungual AR-1-3172/31 in: (KKK) left lateral view, (LLL) dorsal view, (MMM) right lateral view, (NNN) ventral view, and (OOO) proximal view. Pedal ungual AR-1-3181/31 in: (PPP) right lateral view, (QQQ) dorsal view, (RRR) right lateral view, (SSS) ventral view, and (TTT) proximal view. Pedal ungual AR-1-2952/31 in: (UUU) left lateral view, (VVV) dorsal view, (WWW) right lateral view, (XXX) ventral view, and (YYY) proximal view. Pedal ungual AR-1-3291/31 in: (ZZZ) left lateral view, (AAAA) dorsal view, (BBBB) right lateral view, (CCCC) ventral view, and (DDDD) proximal view. Pedal ungual AR-1-3288/31 in: (EEEE) left lateral view, (FFFF) dorsal view, (GGGG) right lateral view, (HHHH) ventral view, and (IIII) proximal view. Pedal ungual AR-1-3182/31 in: (JJJJ) left lateral view, (KKKK) dorsal view, (LLLL) right lateral view, (MMMM) ventral view, and (NNNN) proximal view. Pedal ungual AR-1-3386/31 in: (OOOO) left lateral view, (PPPP) dorsal view, (QQQQ) ventral view, and (RRRR) proximal view. Pedal ungual AR-1-2986/31 in: (SSSS) left lateral view, (TTTT) dorsal view, (UUUU) right lateral view, (VVVV) ventral view, and (WWWW) proximal view. Manual ungual AR-1-3711/31 in: (XXXX) left lateral view, (YYYY) dorsal view, (ZZZZ) right lateral view, (AAAAA) ventral view, and (BBBBB) proximal view.

**Figure 26:**
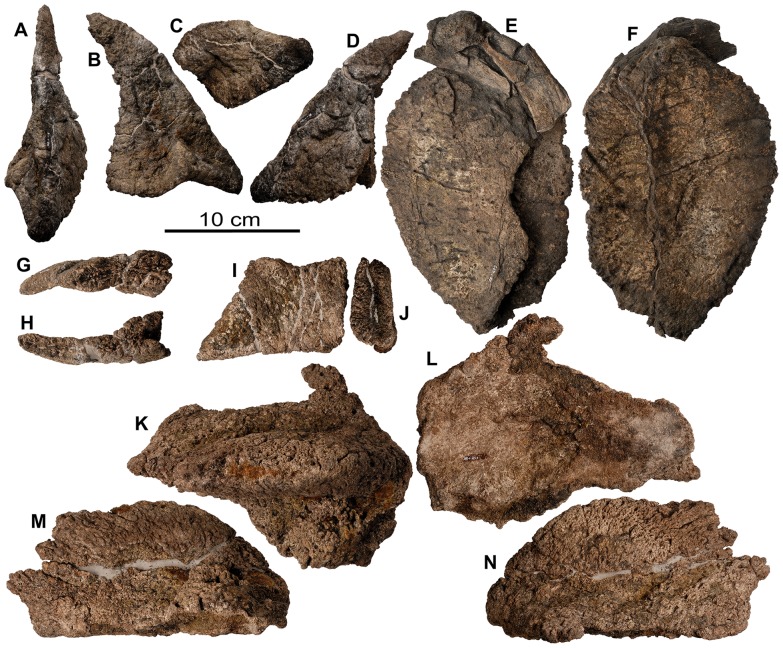
Larger osteoderms of holotype of *Europelta carbonensis* n. gen., n. sp. AR-1/10. Type A dermal armor; cervical or pectoral spine AR-1-128/10 in: (A) anterior view, (B) dorsal view, (C) basal view, and (D) ventral view. Type B dermal armor; caudosacral plate-like osteoderm AR-1-675/10 in: (E) ventral view, and (F) dorsal view. Type A-B dermal armor; distal spine AR-1-444/10 in: (G) anterior view (H) posterior view, (I) dorsal view, and (J) basal view (J). Possible pelvic spine AR-1-653/10 in: (K) dorsal view, (L) ventral lateral, (M)?right lateral view, and (N)?left lateral view.

**Figure 27:**
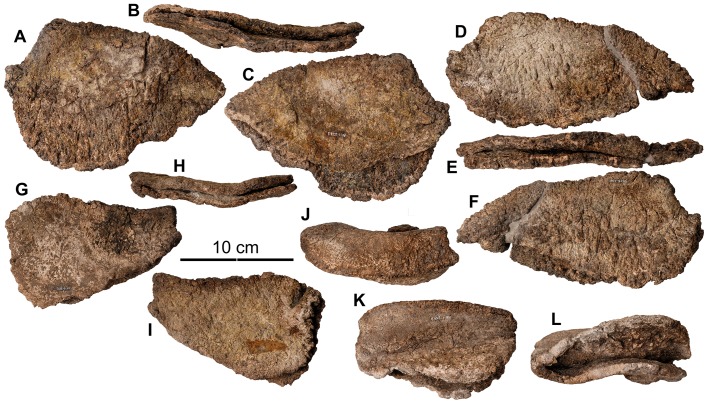
Caudosacral plate-like osteoderms from *Europelta carbonensis* n. gen., n. sp. AR-1/31. Type B dermal armor; caudosacral plate-like osteodrem AR-1-3223/31 in: (A) dorsal view, (B) basal view, and (C) ventral view. Type B dermal armor; caudosacral plate-like osteoderm AR-1-3236/31 in: (D) dorsal view, (E) basal view, and (F) ventral view. Type B dermal armor; caudosacral plate AR-1-3075/31 in: (G) dorsal view, (H) basal view, and (I) ventral view. Type B dermal armor; caudosacral plate-like osteoderm AR-1-3540/31 in: (J) external view, (K) ventral view, and (L) basal view.

**Figure 28:**
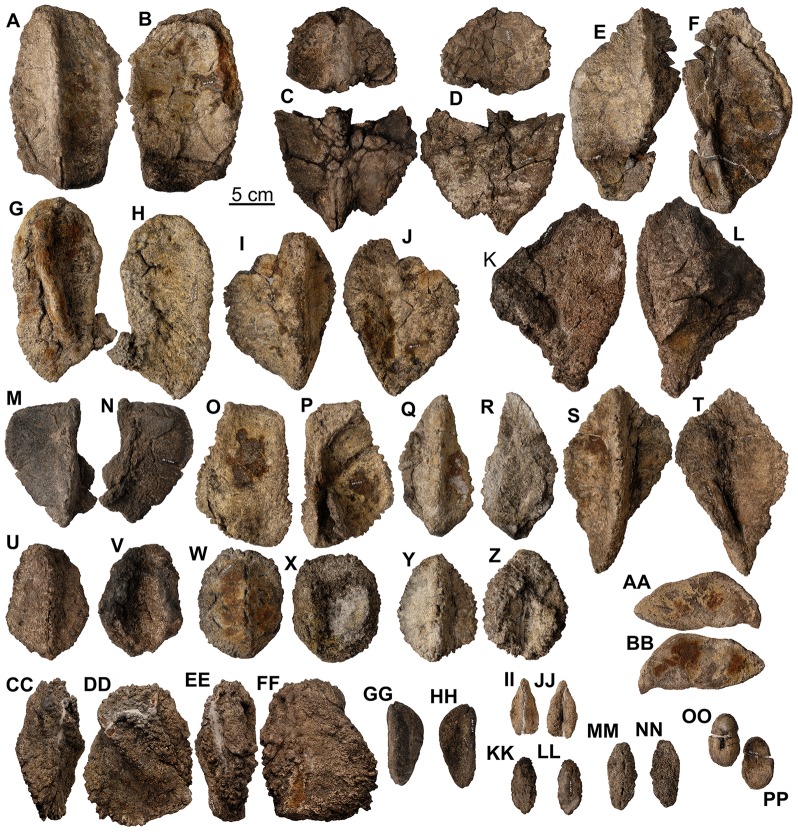
Dorsal osteoderms of holotype of *Europelta carbonensis* n. gen., n. sp. AR-1/10. Type C armor AR-1-467/10 in: (A) external view and (B) basal view. Type C armor AR-1-127/10 in: (C) external view and (D) basal view. Type C-D armor AR-1-461/10 in: (E) external view and (F) basal view. Type C-D armor AR-1-652/10 in: (G) external view and (H) basal view. Type D armor AR-1-553/10 in: (I) external view and (J) basal view (J). Type D armor AR-1-464/10 in: (K) external view and (L) basal view. Type C armor AR-1-4450/10 in: (M) external view and (N) basal view. Type B-C armor AR-1-462/10 in: (O) external view and (P) basal view. Type E armor AR-1-472/10 in: (Q) external view and (R) basal view. Type D-E armor AR-1-651/10 in: (S) external view and (T) basal view. Type F armor AR-1-234/10 in: (U) external view and (V) basal view. Type F armor AR-1-241/10 in: (W) external view and (X) basal view. Type F armor AR-1-659/10 in: (Y) external view and (Z) basal view. Type G armor AR-1-192/10 in: (AA) external view and (BB) basal view. Irregular armor mass AR-1-447/10 in: (CC) lateral view, (DD) external view, (EE) lateral oblique view, and (FF) basal view. Small type F armor AR-1-247/10 in: (GG) external view and (HH) basal view. Small type F armor AR-1-126/10 in: (II) external view and (JJ) basal view. Small type F armor AR-1-496/10 in: (KK) external view and (LL) basal view. Small type F armor AR-1-246/10 in: (MM) external view and (NN) basal view. Small osteoderm AR-1-438/10 in: (OO) external view and (PP) basal view.

**Figure 29:**
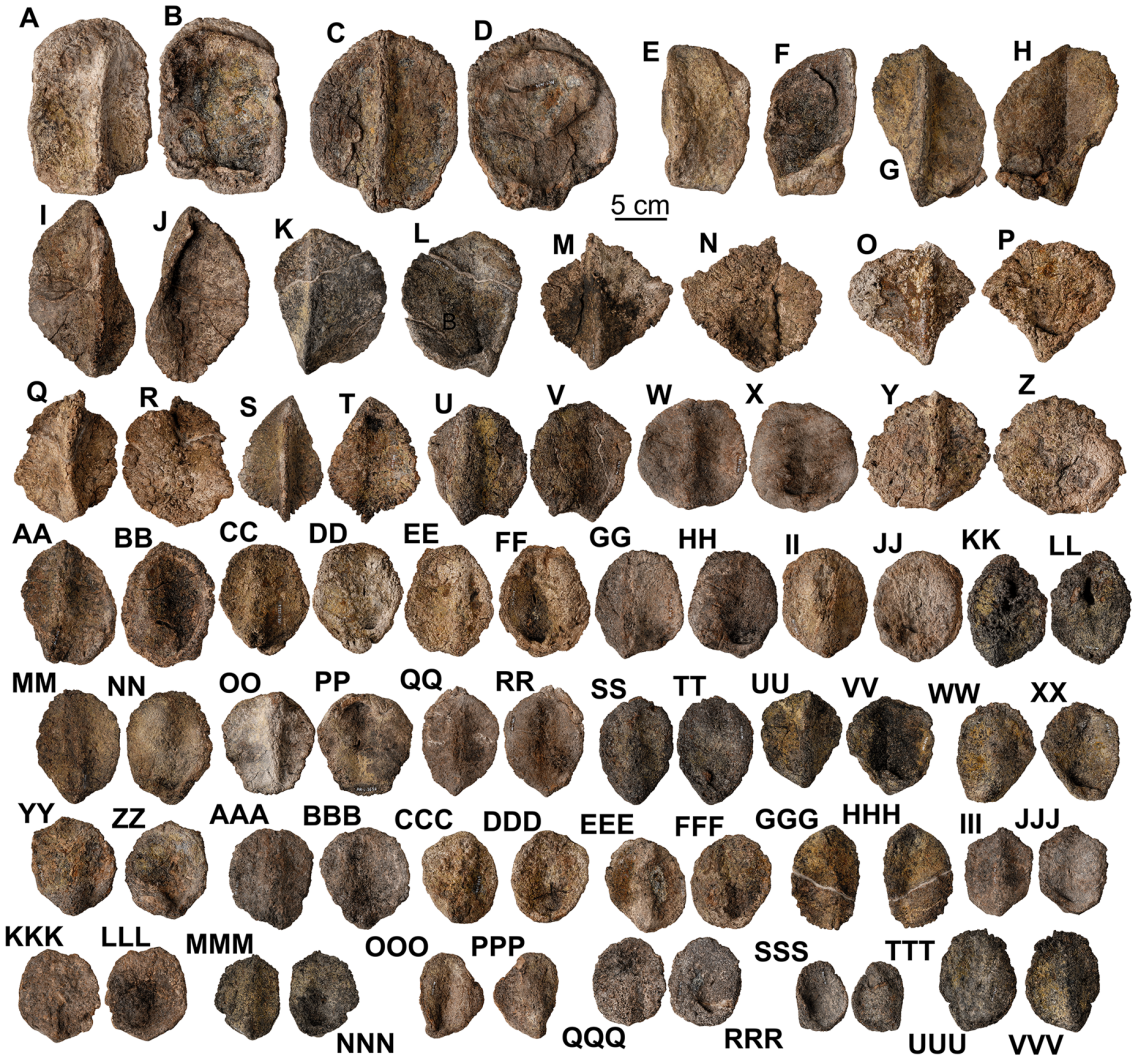
External osteoderms of *Europelta carbonensis* n. gen., n. sp. AR-1/31. Type C armor AR-1-3449/31 in: (A) external view and (B) basal view. Type C armor AR-1-3608/31 in: (C) external view and (D) basal view. Type B-C armor AR-1-3491/31 in: (E) external view and (F) basal view. Type B-C armor AR-1-3492/31 in: (G) external view and (H) basal view. Type D armor AR-1-3590/31 in: (I) external view and (J) basal view. Type D armor AR-1-3587/31 in: (K) external view and (L) basal view. Type D armor AR-1-3438/31 in: (M) external view and (N) basal view. Type D armor AR-1-3390/31 in: (O) external view and (P) basal view. Type D armor AR-1-3030/31 in: (Q) external view and (R) basal view. Type D armor AR-1-3209/31 in: (S) external view and (T) basal view. Type D-F armor AR-1-3572/31 in: (U) external view and (V) basal view. Type F armor AR-1-3681/31 in: (W) external view and (X) basal view. Type F armor AR-1-3340/31 in: (Y) external view and (Z) basal view. Type F armor AR-1-3448/31 in: (AA) external view and (BB) basal view. Type F armor AR-1-3228/31 in: (CC) external view and (DD) basal view. Type F armor AR-1-3447/31 in: (EE) external view and (FF) basal view. Type F armor AR-1-3226/31 in: (GG) external view and (HH) basal view. Type F armor AR-1-3080/31 in: (II) external view and (JJ) basal view. Type F armor AR-1-3576/31 in: (KK) external view and (LL) basal view. Type F armor AR-1-3638/31 in: (MM) external view and (NN) basal view. Type F armor AR-1-3658/31 in: (OO) external view and (PP) basal view. Type F armor AR-1-3683/31 in: (QQ) external view and (RR) basal view. Type F armor AR-1-3573/31 in: (SS) external view (TT) and basal view. Type F armor AR-1-3574/31 in: (UU) external view and (VV) basal view. Type F armor AR-1-3597/31 in: (WW) external view and (XX) basal view. Type F armor AR-1-3610/31 in: (YY) external view and (ZZ) basal view. Type F armor AR-1-3682/31 in: (AAA) external view and (BBB) basal view. Type F armor AR-1-3339/31 in: (CCC) external view and (DDD) basal view. Type F armor AR-1-3180/31 in: (EEE) external view and (FFF) basal view. Type F armor AR-1-3687/31 in: (GGG) external view and (HHH) basal view. Type F armor AR-1-3609/31 in: (III) external view and (JJJ) basal view. Type F armor AR-1-3680/31 in: (KKK) external view and (LLL) basal view. Type F armor AR-1-3684/31 in: (MMM) external view and (NNN) basal view. Small type D armor AR-1-3575/31 in: (OOO) external view and (PPP) basal view. Type F armor AR-1-3074/31 in: (QQQ) external view and (RRR) basal view. Type F armor AR-1-3708/31 in: (SSS) external view and (TTT) basal view. Type F armor AR-1-3720/31 in: (UUU) external view and (VVV) basal view.

**Figure 30:**
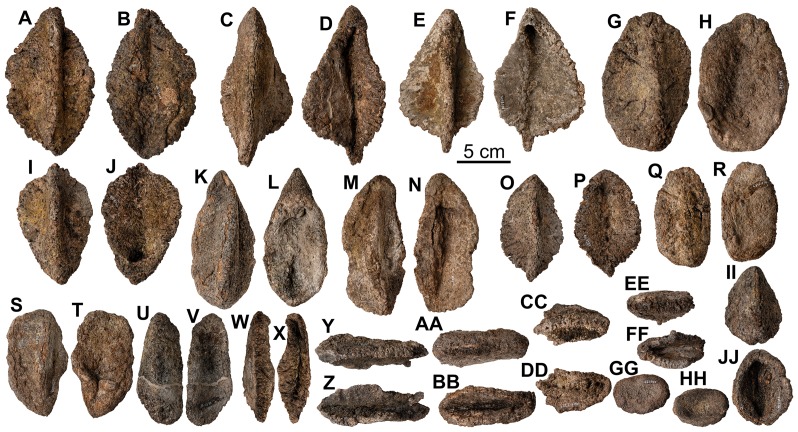
Elongate armor elements of *Europelta carbonensis* n. gen., n. sp. AR-1/31. Type D-E armor AR-1-3024/10 in: (A) external view and (B) basal view. Type D-E armor AR-1-3145/10 in: (C) external view and (D) basal view. Type D-E armor AR-1-3229/10 in: (E) external view and (F) basal view. Type E armor AR-1-3588/31 in: (G) external view and (H) basal view. Type D-E armor AR-1-3207/31 in: (I) external view and (J) basal view. Type E armor AR-1-3216/31 in: (K) external view and (L) basal view. Type E armor AR-1-3242/31 in: (M) external view and (N) basal view. Type E armor AR-1-3208/31 in: (O) external view and (P) basal view. Type E armor AR-1-3494/31 in: (Q) external view and (R) basal view. Type E armor AR-1-3612/31 in: (S) external view and (T) basal view. Type E armor AR-1-3598/31 in: (U) external view and (V) basal view. Type E armor AR-1-3338/31 in: (W) external view and (X) basal view. Type E armor AR-1-3932/31 in: (Y) external view and (Z) basal view. Type E armor AR-1-3611/31 in: (AA) external view and (BB) basal view. Type E armor AR-1-3227/31 in: (CC) external view and (DD) basal view. Type E armor AR-1-3613/31 in: (EE) external view and (FF) basal view. Deeply excavated osteoderm AR-1-3292/31 in: (GG) external view and (HH) basal view. Deeply excavated osteoderm AR-1-3721/31 in: (II) external view and (JJ) basal view.

**Figure 31:**
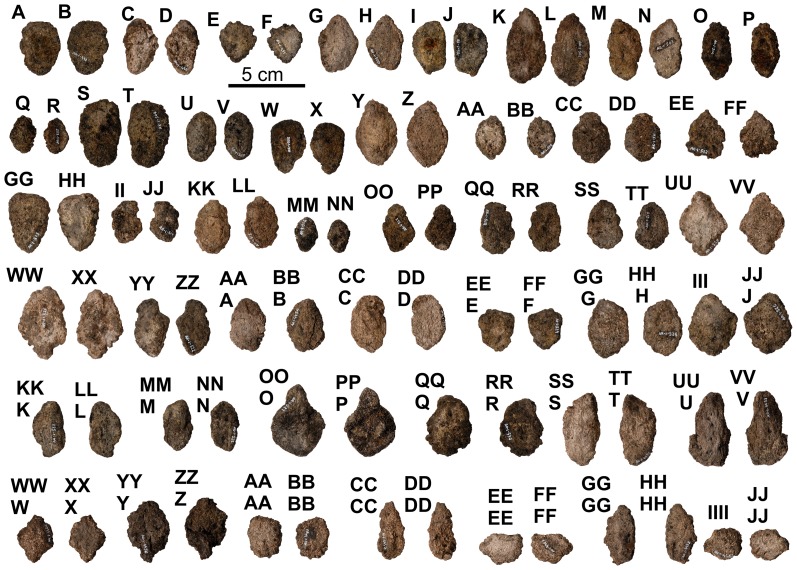
Interstitial Type H armor ossicles of holotype of *Europelta carbonensis* n. gen., n. sp. AR-1/10. Interstitial ossicle AR-1-497/10 in: (A) external view and (B) basal view. Interstitial ossicle AR-1-498/10 in: (C) external view and (D) basal view. Interstitial ossicle AR-1-499/10 in: (E) external view and (F) basal view. Interstitial ossicle AR-1-500/10 in: (G) external view and (H) basal view. Interstitial ossicle AR-1-501/10 in: (I) external view and (J) basal view. Interstitial ossicle AR-1-502/10 in: (K) external view and (L) basal view. Interstitial ossicle AR-1-503/10 in: (M) external view and (N) basal view. Interstitial ossicle AR-1-504/10 in: (O) external view and (P) basal view. Interstitial ossicle AR-1-505/10 in: (Q) external view and (R) basal view. Interstitial ossicle AR-1-506/10 in: (S) external view and (T) basal view. Interstitial ossicle AR-1-507/10 in: (U) external view and (V) basal view. Interstitial ossicle AR-1-508/10 in: (W) external view and (X) basal view. Interstitial ossicle AR-1-509/10 in: (Y) external view and (Z) basal view. Interstitial ossicle AR-1-510/10 in: (AA) external view and (BB) basal view. Interstitial ossicle AR-1-511/10 in: (CC) external view and (DD) basal view. Interstitial ossicle AR-1-512/10 in: (EE) external view and (FF) basal view. Interstitial ossicle AR-1-513/10 in: (GG) external view and (HH) basal view. Interstitial ossicle AR-1-514/10 in: (II) external view and (JJ) basal view. Interstitial ossicle AR-1-515/10 in: (KK) external view and (LL) basal view. Interstitial ossicle AR-1-516/10 in: (MM) external view and (NN) basal view. Interstitial ossicle AR-1-517/10 in: (OO) external view and (PP) basal view. Interstitial ossicle AR-1-518/10 in: (QQ) external view and (RR) basal view. Interstitial ossicle AR-1-519/10 in: (SS) external view and (TT) basal view. Interstitial ossicle AR-1-520/10 in: (UU) external view and (VV) basal view. Interstitial ossicle AR-1-521/10 in: (WW) external view and (XX) basal view. Interstitial ossicle AR-1-522/10 in: (YY) external view and (ZZ) basal view. Interstitial ossicle AR-1-4454/10 in: (AAA) external view and (BBB) basal view. Interstitial ossicle AR-1-523/10 in: (CCC) external view and (DDD) basal view. Interstitial ossicle AR-1-524/10 in: (EEE) external view and (FFF) basal view. Interstitial ossicle AR-1-525/10 in: (GGG) external view and (HHH) basal view. Interstitial ossicle AR-1-526/10 in: (III) external view and (JJJ) basal view. Interstitial ossicle AR-1-527/10 in: (KKK) external view and (LLL) basal view. Interstitial ossicle AR-1-528/10 in: (MMM) external view and (NNN) basal view. Interstitial ossicle AR-1-529/10 in: (OOO) external view and (PPP) basal view. Interstitial ossicle AR-1-530/10 in: (QQQ) external view and (RRR) basal view. Interstitial ossicle AR-1-4459/10 in: (SSS) external view and (TTT) basal view. Interstitial ossicle AR-1-4455/10 in: (UUU) external view and (VVV) basal view. Interstitial ossicle AR-1-4460/10 in: (WWW) external view and (XXX) basal view. Interstitial ossicle AR-1-4456/10 in: (YYY) external view and (ZZZ) basal view. Interstitial ossicle AR-1-4461/10 in: (AAAA) external view and (BBBB) basal view. Interstitial ossicle AR-1-4457/10 in: (CCCC) external view and (DDDD) basal view. Interstitial ossicle AR-1-4462/10 in: (EEEE) external view and (FFFF) basal view. Interstitial ossicle AR-1-4458/10 in: (GGGG) external view and (HHHH) basal view. Interstitial ossicle AR-1-4463/10 in: (IIII) external view and (JJJJ) basal view.

**Figure 32:**
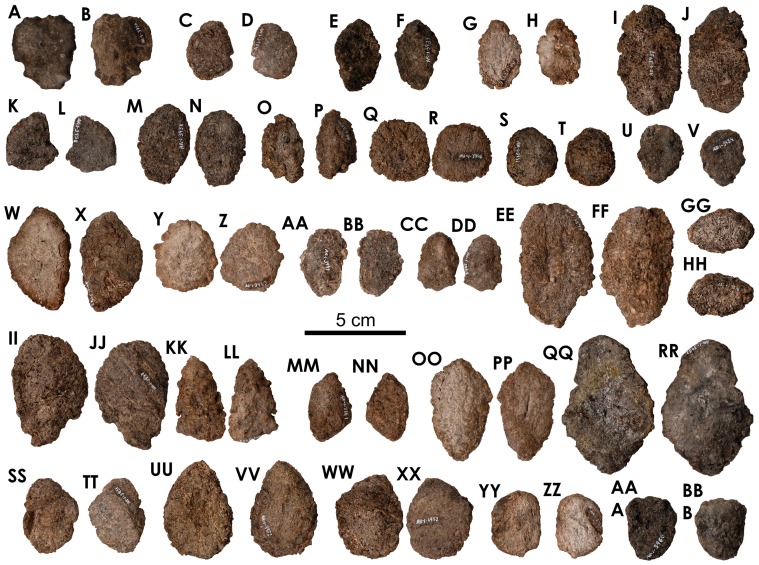
Interstitial Type H ossicles of *Europelta carbonensis* n. gen., n. sp. AR-1/31. Interstitial ossicle AR-1-3933/31 in: (A) external view and (B) basal view. Interstitial ossicle AR-1-3934/31 in: (C) external view and (D) basal view. Interstitial ossicle AR-1-3935/31 in: (E) external view and (F) basal view. Interstitial ossicle AR-1-3936/31 in: (G) external view and (H) basal view. Interstitial ossicle AR-1-3937/31 in: (I) external view and (J) basal view. Interstitial ossicle AR-1-3958/31 in: (K) external view and (L) basal view. Interstitial ossicle AR-1-3938/31 in: (M) external view and (N) basal view. Interstitial ossicle AR-1-3939/31 in: (O) external view and (P) basal view. Interstitial ossicle AR-1-3940/31 in: (Q) external view and (R) basal view. Interstitial ossicle AR-1-/313941 in: (S) external view and (T) basal view. Interstitial ossicle AR-1-3959/31 in: (U) external view and (V) basal view. Interstitial ossicle AR-1-3942/31 in: (W) external view and (X) basal view. Interstitial ossicle AR-1-3943/31 in: (Y) external view and (Z) basal view. Interstitial ossicle AR-1-3944/31 in: (AA) external view and (BB) basal view. Interstitial ossicle AR-1-3945/31 in: (CC) external view and (DD) basal view. Interstitial ossicle AR-1-3946/31 in: (EE) external view and (FF) basal view. Interstitial ossicle AR-1-3076/31 in: (GG) external view and (HH) basal view. Interstitial ossicle AR-1-3947/31 in: (II) external view and (JJ) basal view. Interstitial ossicle AR-1-3948/31 in: (KK) external view and (LL) basal view. Interstitial ossicle AR-1-3949/31 in: (MM) external view and (NN) basal view. Interstitial ossicle AR-1-3950/31 in: (OO) external view and (PP) basal view. Interstitial ossicle AR-1-3957/31 in: (QQ) external view and (RR) basal view. Interstitial ossicle AR-1-3951/31 in: (SS) external view and (TT) basal view. Interstitial ossicle AR-1-3952/31 in: (UU) external view and (VV) basal view. Interstitial ossicle AR-1-3953/31 in: (WW) external view and (XX) basal view. Interstitial ossicle AR-1-3956/31 in: (YY) external view and (ZZ) basal view. Interstitial ossicle AR-1-3960/31 in: (AAA) external view and (BBB) basal view.

**Figure 33:**
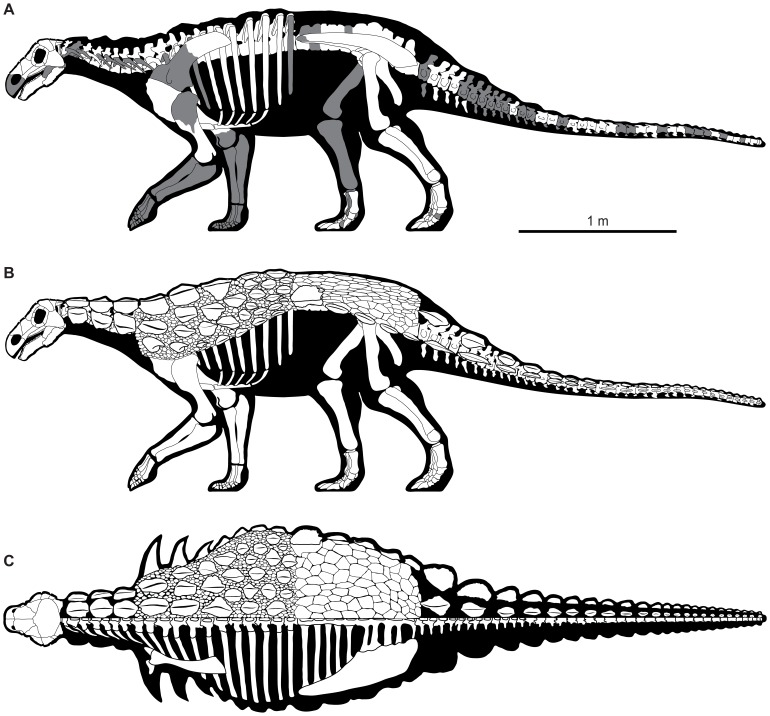
Skeletal reconstruction of *Europelta carbonensis* n. gen., n. sp. Skeletal reconstruction in: (A) lateral view with unknown parts of the skeleton shaded in gray, (B) lateral view with hypothetical distribution of the armor indicated, and (C) dorsal view with hypothetical distribution of the armor on right side of body indicated.

### Etymology

The specific name “*carbonensis*” from the coal, is in honor of access to the fossil locality in the Santa María coal mine provided by Sociedad Anónima Minera Catalano-Aragonesa (SAMCA Group), which has been extracting coal in Ariño (Teruel) since 1919.

### Holotype

AR-1/10, a disarticulated partial skeleton reposited at Fundación Conjunto Paleontológico de Teruel-Dinópolis/Museo Aragonés de Paleontología (FCPTD/MAP). The holotype consists of: a mostly complete skull (AR-1-544), isolated left and right nasals (AR-1-133, and AR-1-639), a dentary fragment (AR-1-362), 15 isolated teeth (AR-1-323 to AR-1-325, AR-1-343, AR-1-358, AR-1-417, AR-1-418, AR-1-423, AR-1-424, AR-1-428, AR-1-454, AR-1-482, AR-1-563, AR-1-564 and AR-1-567), an atlas (AR-1-649), five cervical vertebrae (AR-1-431, AR-1-449, AR-1-533, AR-1-637, AR-1-650), two cervical ribs (AR-1-450, AR-1-4452), AR-1-638 (possibly the first dorsal vertebrae), seven more posterior dorsal vertebrae (AR-1-154, AR-1-155, AR-1-322, AR-1-430, AR-1-448, AR-1-478, AR-1-535, AR-1-556), a section of synsacrum (AR-1-154), three isolated dorsal ribs (AR-1-331, AR-1-333, AR-1-476), seven dorsal rib fragments (AR-1-339, AR-1-341, AR-1-427, AR-1-534, AR-1-641, AR-1-642, AR-1-676), three caudal vertebrae (AR-1-562, AR-1-635, AR-1-636), four chevrons (AR-1-560, AR-1-561, AR-1-569, AR-1-4451), a coracoid with a small portion of scapula (AR-1-657), a scapular blade fragment (AR-1-429), two xiphosternal plates (AR-1-252, AR-1-4675), two partial humeri (AR-1-327, AR-1-655), a right ilium-ischium-pubis (AR-1-479), a left ischium-pubis (AR-1-129), and 70 osteoderms (AR-1-126 to AR-1-128, AR-1-192, AR-1-234, AR-1-241, AR-1-246, AR-1-247, AR-1-272, AR-1-276, AR-1-438, AR-1-444, AR-1-447, AR-1-461, AR-1-462, AR-1-464, AR-1-467, AR-1-472, AR-1-496 to AR-1-530, AR-1-553, AR-1-651 to AR-1-653, AR-1-659, AR-1-675, AR-1-4450, AR-1-4454 to AR-1-4463).

### Paratype

AR-1/31, a partial skeleton deposited at Fundación Conjunto Paleontológico de Teruel-Dinópolis/Museo Aragonés de Paleontología (FCPTD/MAP). The paratype consists of a partial left jaw with dentary and surangular (AR-1-3698) and isolated angular (AR-1-2945), 10 teeth (AR-1-3432, AR-1-3495, AR-1-3524, AR-1-3650, AR-1-3699 to AR-1-3701, AR-1-3705, AR-1-3706, AR-1-3961), five cervical vertebrae (AR-1-3586, AR-1-3632, AR-1-3657, AR-1-3671, AR-1-3676), nine dorsal vertebrae (AR-1-3489, AR-1-3586, AR-1-3633, AR-1-3662, AR-1-3672 to 3675, AR-1-3677, AR-1-3704), three to four? dorsosacral vertebrae (AR-1-3450, AR-1-3451), a sacrum (AR-1-3446), a caudosacral vertebra (AR-1-3512), two sacral rib fragments (AR-1-3452, AR-1-3460), 14 caudal vertebrae (AR-1-2950, AR-1-3204, AR-1-3206, AR-1-3243, AR-1-3265, AR-1-3348, AR-1-3398, AR-1-3478, AR-1-3615, AR-1-3616, AR-1-3714 to 3717), a right ilium (AR-1-3490), two left ilium fragments (AR-1-3521, AR-1-3571), two ischia with fused pubes (AR-1-3648, AR-1-3649), a right femur (AR-1-3244), a right tibia (AR-1-3237), a right fibula (AR-1-3238), a calcaneum (AR-1-3239), four metatarsals (AR-1-3100, AR-1-3173, AR-1-3233, AR-1-3234), eight phalanges (AR-1-3032, AR-1-3066, AR-1-3174, AR-1-3179, AR-1-3324, AR-1-3234, AR-1-3292, AR-1-3356), nine unguals (AR-1-2952, AR-1-2986, AR-1-3172, AR-1-3181, AR-1-3182, AR-1-3288, AR-1-3291, AR-1-3386, AR-1-3711), and 90 osteoderms (AR-1-3024, AR-1-3030, AR-1-3074 to AR-1-3076, AR-1-3080, AR-1-3145, AR-1-3159, AR-1-3180, AR-1-3207 to AR-1-3209, AR-1-3216, AR-1-3223, AR-1-3226 to AR-1-3229, AR-1-3292, AR-1-3236, AR-1-3242, AR-1-3338 to AR-1-3340, AR-1-3390, AR-1-3438, AR-1-3447 to AR-1-3449, AR-1-3491, AR-1-3492, AR-1-3494, AR-1-3506, AR-1-3540, AR-1-3572 to AR-1-3576, AR-1-3587, AR-1-3588, AR-1-3590, AR-1-3597, AR-1-3598, AR-1-3608 to AR-1-3613, AR-1-3638, AR-1-3658, AR-1-3680 to AR-1-3684, AR-1-3687, AR-1-3708, AR-1-3720, AR-1-3721, AR-1-3932 to AR-1-3960).

### Locality and Horizon

The type locality, Fundación Conjunto Paleontológico of Teruel-Dinópolis locality AR-1, is located east of Ariño, Teruel Province, Spain. The fossil horizon is below the lowest mineable coal seam at Sociedad Anónima Minera Catalano-Aragonesa Group's Ariño coal mine in a plant debris bed in the lower Escucha Formation [42]. The paratype AR-1/31 was located 200 m laterally from the holotype AR-1/10 in the same bed. Pyrite is common within the bone and the surrounding sediment of the bonebed, common also in plant debris beds in the older Wessex Formation on the Isle of Wight [58].

### Age

Elsewhere, the Escucha Formation has been interpreted as late Aptian to early Albian in age based on nanofossils, planktonic foraminifera, dinoflagellates and palynomorphs [50], [52]. An analysis of the palynomorphs, ostracods, and charophytes from AR-1 indicates that the site is completely of early Albian age [57].

### Diagnosis

The quadrate is shorter and mediolaterally wider than in any other ankylosaur. The posterior margin of the skull is concave in dorsal view. The sacrum is arched dorsally about 55° in lateral view. The pubis is fully and uniquely fused to the ischium with a slot-shaped foramen between the post-pubic process and the position of the pubic peduncle forming an ischiopubis. The tibia is longer relative to the length of the femur (90%) than in other ankylosaurs for which these proportions are known. Laterally compresed, flanged osteoderm with a flat plate-like base is present anteriorly on the pelvic shield.

## Description and Comparisons

### Skull

The skull (AR-1-544/10) was lying on its dorsal surface and is moderately well preserved although distorted through compaction (Fig. 7). The palate is crushed in toward the skull roof, resulting in the medial rotation of both maxillae with the posterior teeth displaced into the posterior palate. The sheet-like palatal bones are highly fragmented. The braincase is crushed along the plane of the cranial nerve openings and the fenestra ovalis completely obscures them. Unexpectedly, the right quadrate (Fig. 8 H–J) and associated portion of the palate was dislodged from the skull and subsequently crushed across the ventral side of the basicranium. This gives the impression that these bones had been expelled from inside the skull prior to compaction. Both the left and right nasals were separated from the skull and the premaxillae (whereas possibly present upon discovery) have not been identified.

The skull has a minimum length of 370.3 mm from the anterior end of the maxillae to the rear margin of the squamosals. The skull has a maximum width of 299.1 mm at the orbits and narrows to 203.7 mm at the posterior end of the skull at the squamosals, giving the skull the “pear-shaped” dorsal profile characteristic of derived nodosaurids [70], [71]. Although tapering posteriorly, there is no distinct post-temporal notch as in polacanthids and other nodosaurids [63].

The maxillae (Fig. 7 D–F) are irregularly sculptured externally with a flattened, horizontally oriented buccal recesses that are inset approximately 2 cm. The anterior margin of the maxilla appears to form the posterior margin of a relatively simple naris relative to derived nodosaurids and ankylosaurids. Medially, there is no evidence that the maxilla formed a portion of a secondary palate. The tooth row was arched ventrally with an estimated 22–25 alveoli increasing in size posteriorly as in *Edmontonia* [72]. In ventral orientation, the tooth rows are only moderately deflected medially, such that the palate would not have had a pronounced hourglass appearance typical of derived nodosaurs such as *Pawpawsaurus*, *Edmontonia*, and *Panoplosaurus* [73]–[75]. However, it is not dissimilar from that of the primitive nodosaurid *Silvisaurus* [76], [77].

The nasals (AR-1-133/10, AR-1-639/10) are relatively large and subrectangular, tapering somewhat anteriorly (Fig. 8 A–D). Both nasals extend laterally from their relatively straight, unfused midline suture before flexing down to a sutural contact with the maxillae that extends for most of their length. When rearticulated onto the skull, they appear to fit well, despite the skull's distortion. Most ankylosaurs have fused nasals except the nodosaurids *Silvisaurus* [76], [77] and *Niobrarasaurus* [78], although the nasals are unknown in European nodosaurids [24], [32], [33]. A distinct tongue-like process projects from the nasal's posterior margin and would have overlapped the frontals. The external surface is lightly textured and the internal surface is relatively smooth, suggesting the narial passage was large and simple, rather than convolute as in derived nodosaurids and ankylosaurids [79], [80].

The orbits are somewhat crushed and the sutures of the bones surrounding them are obscured by fusion. The orbits are subrectangular in shape, are slightly more elongate anteoposteriorly and are directed anterolaterally. The prominent and evenly rounded suborbital horn is formed mostly from the quadratojugal posterior to the ventral margin of the orbit, as in most derived ankylosaurs [81], [82] and unlike that in polacanthids such as *Mymoorapelta, Gargoyleosaurus*, and *Gastonia* where the suborbital horn is below the orbit and is formed exclusively by the jugal [83]–[85]. The suborbital horn appears to be unornamented and hides the head of the quadrate in lateral view.

The lateral wall of the skull extends posteriorly behind orbit with a dorsoventally wide posterior notch, such that the lower temporal opening is just visible in lateral view. There is no lateral wall of skull behind the orbits in polacanthids [70], [81] and most nodosaurids other than *Peloroplites* [86], *Silvisaurus* [76], *Struthiosaurus transylvanicus* [22], [23] and one specimen from the Dinosaur Park Formation assigned to *Edmontonia* (ROM 1215) [88], although in these taxa the lower temporal opening is still visible in lateral view as in *Europelta*. The lower temporal opening is completely obscured in lateral view in *Cedarpelta* [84], [86], *Shamosaurus* [89]–[91], *Gobisaurus*, [92] *Zhongyuansaurus* [93] and all derived ankylosaurids.

Although the palate is fragmented and crushed along the internal surface of the skull roof, the fragments of the vomer suggest it did not extend ventrally to the level of the tooth row. Additionally, the broad sheet-like pterygoids appear to have been flexed nearly dorsally against the anterior portion of the basicranium as in nodosaurids and not like the open transversely oriented pterygoids characteristic of ankylosaurids or polacanthids [94].

The posterolateral margin of the pterygoid is fully fused to the quadrate. There is a sutural contact between the straight, nearly vertical quadrates and the quadratojugal laterally. The quadrates are wide transversely and thin rostrocaudally as compared to the mediolaterally narrower quadrates of other ankylosaurs [82]. The contact with the squamosal is also transversely wide, unlike the narrow, rounded contact seen in many ankylosaurs such as *Mymoorapelta* (Kirkland, pers. obs.) and *Cedarpelta* [63], [86]. The mandibular articulation is proportionally wider than in any other ankylosaur examined as a part of this study and the medial condyle larger than the lateral condyle. The ratio of mediolateral quadrate width to dorsoventral quadrate length is 0.77 (94 mm/122 mm). The anteropostior length of the quadrate condyle is 31 mm. There is no fusion between the quadrates and the paroccipital processes.

Vertical compaction has obscured the posterior view of the skull, in particular the foramen magnum and the supraoccipital. However, even with compaction it is apparent that in occipital view the skull was subrectangular and wider than tall as in *Gargoyleosaurus*, *Gastonia*, and most other derived anklylosaurs, and unlike the narrow, highly arched occipital region of *Struthiosaurus* [22]. The paroccipital processes extend horizontally lateral to the foramen magnum and then flare dorsoventrally by approximately 100% of their minimum widths. They angle posteriorly at about 30 degrees when viewed ventrally (Fig. 7 F). In morphology and orientation, they are most similar to those in *Gargoyleosaurus* [95] although ventral twisting is not present. In most other ankylosaurs, the paroccipital processes extend straight laterally [81], [96] or may be flexed ventrally as in *Gastonia* [83]. A triangular wedge of bone of unknown identity is fused to the anterior ventrolateral margin of the paroccipital, separating it from the quadrate.

The subspherical occipital condyle (Fig. 7 B, F) has a width of 59.4 mm and height of 46.5 mm and lacks a distinct neck to separate it from the rest of the basicranium. Although no cranial sutures are visible, the occipital condyle does appear to be composed exclusively of the basioccipital. It is similar in overall morphology to that of the basal ankylosaurid *Cedarpelta* [88] except that the occipital condyle angles somewhat ventrally, but not as much as in more derived nodosaurids [71], [82]. The ventral surface of the relatively elongate basioccipital is broadly convex. Again, as in *Cedarpelta* [88], there are no distinct, separate basal tubera between the basioccipital and the short basisphenoid, but instead there is a prominent transverse flange extending across the ventral surface of the basicranium along the line of this suture. The pterygoid processes appear to be short, but are completely obscured by crushed pterygoids bone fragments that wall off the anterior part of the braincase as in most nodosaurids.

The skull roof (figs. 7 C, 9 A) is roughened texturally by remodeling of the bone surface as in *Cedarpelta*, the nodosaurids *Sauropelta* and *Peloroplites*, and the shamosaurine-grade ankylosaurids *Shamosaurus* and *Gobisaurus* [81], [86], [88]. *Europelta* differs from these specimens in that some of the margins of the scale impressions on the skull roof are visible, as seen in *Edmontonia*, *Panoplosaurus* and *Struthiosaurus* [22], [77]. These scale margins are represented by shallow grooves that are difficult to see relative to the textured surface of the skull and the cracks in the bone due to compaction. These grooves are particularly evident along the lateral margins of the skull roof above the orbit. An extensive median scale appears to have covered much of the central portion of the skull between and posterior to the orbits on the frontals and parietals as other nodosaurids [63], [82]. There does not appear to be any distinct nuchal ornamentation. The skull is thickened above the orbit, but there is not a distinct supraorbital boss, a condition similar to *Peloroplites*, *Cedarpelta*, *Shamosaurus*, and *Gobisaurus* [86], [88]–[90], [92]. Narrow grooves along the margin of the skull in this area above the orbits suggest that a particularly robust pair of scales were present in this area as indicated by a deep groove bisecting this ornamented area directly above the orbit. Weak grooves delineate a small scale without underlying ornamentation separating the posterior supraorbital scale from the squamosal horn forming the posteriolateral margin of the skull roof. The squamosal horn is ornamented by narrow grooves radiating from its apex onto the skull roof. Grooves on the anterolateral sides of the fronto-parietal scale appear to delineate two scales between the anterior supraorbital scales. Unfortunately, no distinctive scale boundaries are recognizable on the nasals, although the dorsal surfaces of the nasals are textured. Several elongate scales rimmed the lateral raised margin around the orbit.

In dorsal view, the posterior margin of the skull is concave, whereas it is nearly straight or convex in all other nodosaurids. This reflects the posterior angulation of the paraoccipital processes and the squamosal horns. Interestingly, the occipital condyle is barely visible, though not completely obscured in dorsal view. There is no evidence of any distinct nuchal sculpturing. The skull roof is relatively flat but a slight dome may have been present prior to crushing. However, it is clear that the skull roof is not as highly domed as in many other nodosaurids, such as *Struthiosaurus* [22], [26].

Attempts were made to image the skull using X-ray photography and CT scanning. The abundance of pyrite present in the skull (Fig. 4E) presents a strong limitation in the use of these techniques as pyrite is opaque to X-rays.

### Mandible

A small dentary fragment extending for only four complete alveolae (AR-1-133/10) was preserved from the holotype skeleton (Fig. 8 E–G). However, a robust left dentary and splenial are preserved together (AR-1-3698/31) from the paratype specimen (Fig. 10 A–E). The splenial is not in its posteriomedial position relative to the dentary, but is fused across the posterior portion of the tooth row transversely. Additionally, an isolated left angular with a distinct highly sculptured scale along its ventral margin (AR-1-2945/31), was recovered (Fig. 10 F, G).

The dentary is 184.7 mm long with a minimum of 21 tooth positions, with no possibility of more than two unpreserved alveoli as determined by the position of the suture with the angular and surangular. As with the maxillary teeth, the alveoli are more than twice as large posteriorly. There is only 1.5 cm between the anteriormost alveoli and the symphysis, suggesting that there may have been premaxillary teeth as at least nine anterior teeth would have been positioned to oppose the premaxilla. The primitive ankylosaurs *Sarcolestes* [34], [98], *Gargoyleosaurus*, [85], *Silvisaurus* [76], *Animantarx* [97], *Sauropelta* [99], *Anoplosaurus* [17], *Hungarosaurus* [33] and *Struthiosaurus* [22] have a short anterior diastema, and thus a narrow predentary, whereas this diastema is longer in ankylosaurs with wide predentaries. However, the symphysis in *Europelta* is robust and dorsoventrally deeper (45.0 mm deep and 29.00 mm across) than in ankylosaurs [82], and is most similar to the deep symphysis of *Hungarosaurus* [32], further suggesting a reduced predentary with a rudimentary ventral process. The symphysis is marked by two deep anteroposteriorly directed grooves. A row of foramina extends posteriorly on the lateral surface of the dentary from just dorsal to the buccal recess to the notch for the surangular, whereas nutritive foraminae are not clearly visible ventral to the alveolae on the medial side of the dentary as in other ankylosaurs. The recessed tooth row is deflected medially and forms a convex arch in lateral view. The dentary of *Hungarosaurus* is deeper dorsoventrally than that of *Europelta* [33].

The splenial (Fig. 10 A-D) is a thin bone with a convex ventral margin 156.6 mm long that contacts the angular. It has the appearance of an obtuse triangle in medial view. There is large, well-developed intermandibular foramen (7 mm long and 5.3 mm wide) 50 mm from its anterior end.

The angular (Fig. 10 F, G) has a maximum length of 175 mm. The lateral margin is highly rugose, because the bone is textured and remodeled to support a large scale, extending about 10–12 mm ventral to the ventral margin of the angular for most of its length. A distinct ridge marks the dorsal limit of the mandibular ornament medially, where it is in contact with the ventral margin of the splenial. Dorsal to this contact the bone is smooth. The ventral extent of the textured bone supporting the mandibular scale is similar to that observed in ankylosaurids such as *Euoplocephalus* [95] and *Minataurasaurus* [100], rather than the more lateral orientation found in *Gargoyleosaurus* [93] and in nodosaurids like *Sauropelta* [99] and *Panoplosaurus* [101].

### Teeth

A large number of teeth are preserved from both the holotype AR-1/10 (20+) and the paratype AR-1/31 (15+) although many have drifted away from the alvaeolae. We assume that the teeth associated with the holotype pertain to the maxilla (several are preserved in the palate and in the maxilla) and those of the paratype pertain to the dentary (several are preserved in the dentary). In general, the cutting surfaces of the teeth are not well preserved, but a few exceptions exist. Wear facets were not observed on any of the teeth. The roots for both dentary and maxillary teeth are swollen lingually, are three to four times the length of the crowns, and are subquadrate in cross-section. One small tooth (AR-1-343/10) is more highly asymmetrical mesiodistally and may represent a premaxillary tooth (Fig. 11 L, M).

The isolated maxillary teeth (Fig. 11 A–K, N–FF) have a weakly developed labial cingulum and a strongly developed lingual cingulum. The best preserved right tooth AR-1-324/10 is 11.50 mm wide, 9.99 mm tall with seven to eight mesial denticles and five to six distal denticles (Fig. 11 A–E). A large right tooth AR-1-564/10 is 17.23 mm wide and 12.95 mm tall with eight to nine mesial denticles and ∼six to seven distal denticles (Fig. 11 V–Z).

The isolated dentary teeth (Fig. 11 GG–FFF) are identical to the maxillary teeth and have a weak lingual cingulum and a strongly developed labial cingulum. The best preserved tooth AR-1-3700/31 is 14.03 mm wide and 12.69 mm tall with eight to nine mesial denticles and six to seven distal denticles (Fig. 11 LL–PP). The largest dentary tooth AR-1-3650/31 is 16.58 mm wide and 13.50 mm tall (Fig. 11 GG–KK).

With their relatively large size and well-developed cingula, the teeth of *Europelta* are most comparable to those of other nodosaurids [72]. They similar to the teeth of *Cedarpelta*, *Sauropelta* [34], [97], [102], *Edmontonia* and *Panoplosaurus* [72], but are not as high crowned as in the Jurassic ankylosaurs *Sarcolestes* and *Priodontognathus* [103], the Jurassic polacanthids *Gargoyleosaurus* [93] and *Mymoorapelta* (Kirkland, pers. obs.), the nodosaurids *Peloroplites* [84] or *Hungarosaurus* [33]. Additionally, the large teeth of *Gobisaurus* are more inflated labiolingually than in *Europelta* and other ankylosaurs. The teeth of *Gastonia* and putative *Polacanthus* teeth are also inflated, but are smaller proportionally [83], [103]. The teeth of *Europelta* differ from an isolated tooth from the Cenomanian of France which is about half the size, and proportionally is longer mesiodistally with more deeply divided denticles forming ridges on the labiolingual surfaces of the tooth [104]. Likewise, lower Cenomanian teeth assigned to “*Acanthopholis*” have more deeply divided denticles in what is a proportionally taller tooth [17]. The teeth of *Struthiosaurus languedocensis* [31] from the lower Campanian of France also differ in size and in having longer, lower tooth crowns.

### Axial skeleton

There are numerous ribs and vertebrae preserved from the holotype (AR-1/10) and the paratype specimen (AR-1/31). Vertebral measurements are presented in Table 1.

**Table 1:** Measurements of *Europelta* vertebrae.

				*Europelta* VERTEBRAL MEASUREMENTS IN MM							
	Anterior		Posterior		Overall	Neural	Neural	Total	Neural	Transverse	Transverse
	Centrum Face		Centrum Face		Centrum	Canal	Canal	Vertebral	Spine	Processes	Processes
	Width	Height	Width	Height	Length	Width	Height	Height	Height	Width	Length
									(above canal)		
AR/10											* estimated
**Cervical Vertebrae**											
AR-1-431	109.2	78.8	-	-	*85.2	30.6	30.6	186.1	86.4	203.8	79.2
AR-1-449	100.1	74.3	-	-	66.3	31.6	22.4	185.5	90.9	198.2	61.1
AR-1-533	94.9	69.9	-	-	*81.7	*23.1	*22.9	*218.9	*133.2	*203.1	72.5
AR-1-637	*81.5	*60.5	*78.3	*60.5	*96.8	*25.8	*17.1	-	-	-	47.3
AR-1-638	93.1	68.9	*85.0	*73.8	75.6	20.6	30.3	-	-	*160.2	86.7
AR-1-649	73.2	70.1	99.9	61.2	62.0	28.8	19.1	-	-	-	77.4
AR-1-650	*81.4	*57.7	80.6	62.5	*61.0	*26.8	*14.4	122.5	*56.0	*104.1	*29.0
**Dorsal Vertebrae**											
AR-1-154	-	-	-	-	79.4	-	-	-	-	-	-
AR-1-155	*60.0	*69.8	75.5	*68.4	*79.2	-	-	*159.5	129.3	-	-
AR-1-322	89.9	76.3	94.6	78.9	82.5	14.8	24.0	-	133.1	-	*85.2
AR-1-430	91.4	77.7	97.5	83.6	84.6	*20.1	*25.2	222.9	-	*175.8	*76.5
AR-1-448	90.3	78.5	95.5	79.0	90.7	*16.8	*23.7	-	-	*120.0	*73.3
AR-1-478	91.4	78.2	94.8	84.0	*86.1	16.7	22.1	219.8	139.2	114.4	85.7
AR-1-535	98.5	81.7	92.8	*82.0	93.5	22.4	26.3	239.9	-	142.5	91.2
AR-1-556	*88.4	*79.2	*83.0	*76.3	*70.7	*21.0	*27.4	-	-	-	*89.8
**Caudal Vertebrae**											
AR-1-562	76.2	73.8	81.6	79.3	72.4	14.3	26.0	178.8	85.8	193.9	64.8
AR-1-635	82.2	79.8	92.4	92.1	79.4	19.4	26.7	192.2	80.7	240.6	74.0
AR-1-636	82.7	80.7	89.1	94.2	*66.2	*23.10	*21.3	*193.0	*88.8	*211.3	77.4
AR/31											
**Cervical Vertebrae**											
AR-1-3632	*76.5	*63.4	*66.0	*63.9	*52.9	*9.0	*18.5	*154.8	*68.2	*139.6	*60.6
AR-1-3657	67.9	53.5	76.0	-	*51.8	13.7	21.1	-	-	*151.6	50.1
AR-1-3662	69.1	60.3	*67.1	60.2	*53.3	-	-	-	-	-	-
AR-1-3671	*69.0	*49.5	*78.0	*52.4	*53.9	*25.2	*11.8	*134.1	*57.3	*120.5	*41.3
AR-1-3676	*52.1	*55.6	*60.3	*60.0	*60.4	*8.8	*19.5	*136.1	*51.1	*89.8	*26.9
**Dorsal Vertebrae**											
AR-1-3489	65.6	59.3	65.0	61.8	79.0	*12.0	*15.1	178.9	105.0	-	68.0
AR-1-3586	75.6	61.6	72.7	61.8	54.6	14.3	19.6	157.5	74.7	140.9	62.9
AR-1-3633	76.4	60.1	67.0	58.6	62.7	13.1	18.9	178.5	94.7	*139.1	73.2
AR-1-3672	68.7	52.7	77.1	57.8	73.5	-	-	-	-	-	-
AR-1-3673	66.6	60.5	66.5	55.4	72.5	*11.8	*15.5	-	-	*119.6	58.0
AR-1-3674	*59.1	*65.7	*53.7	*63.8	*72.9	-	-	*168.5	*88.7	-	85.1
AR-1-3675	*64.6	56.9	66.7	63.2	66.3	15.9	22.3	*171.8	*104.4	*133.9	74.2
AR-1-3704	67.0	62.8	*64.7	*59.9	79.1	-	*14.4	-	-	*154.6	63.3
**Caudal Vertebrae**											
AR-1-2950	31.1	25.3	28.7	24.2	50.2	6.1	4.9	37.0	9.0	-	-
AR-1-3204	-	-	-	-	-	-	-	-	-	49.8	-
AR-1-3206	39.0	29.5	35.4	30.0	50.6	*8.04	*7.0	-	-	-	-
AR-1-3243	43.0	38.8	38.7	31.7	51.0	5.3	6.0	47.3	20.7	-	-
AR-1-3265	45.1	*30.0	45.2	32.0	52.6	-	-	-	-	-	-
AR-1-3348	*60.7	49.5	*55.3	*45.5	*53.7	-	-	73.1	15.1	*99.0	38.2
AR-1-3398	42.5	34.6	32.4	35.3	51.9	3.3	6.2	50.1	13.2	42.5	-
AR-1-3478	48.9	34.8	46.7	38.2	52.2	4.8	8.0	52.2	-	46.1	-
AR-1-3615	51.4	37.1	49.0	26.1	56.1	6.5	11.2	47.7	*8.4	-	-
AR-1-3616	*48.9	*42.8	*45.2	*39.7	*56.9	-	-	*58.9	-	49.3	-
AR-1-3714	30.2	23.7	-	-	42.1	5.7	4.8	-	-	-	-
AR-1-3715	-	-	25.6	22.2	37.7	-	-	31.6	-	-	-
AR-1-3716	-	-	*48.1	43.9	-	-	-	*70.1	15.5	-	-
AR-1-3717	61.4	40.0	58.1	39.6	54.2	7.9	*5.1	-	-	95.4	29.7

The complete atlas (AR-1-649/10) from the holotype has a total width of 195.6 mm (Fig. 12 A–F). The neural arch is divided dorsally with the left side fused to the centrum and the right side unattached. The anterior face of the atlantal intercentrum is 73.7 mm wide by 71.7 mm tall and its posterior face is 99.9 mm wide by 61.2 mm tall with a length of 62.0 mm. The axis is not present in either associated skeleton.

There are five post-axis cervical vertebrae (AR-1-431/10, 449, 533, 637, 650) preserved from the holotype skeleton (Fig. 12 I– II) and five from the paratype skeleton; of which four are illustrated (AR-1-3586/31, 3632, 3671, and 3676) (Fig. 13). Overall, they are typical of most other described ankylosaur cervical vertebrae. The centra are amphicoelus, wider than tall, anterorposteriorly short, and medially constricted. Anterior and mid-cervical vertebrae have the anterior faces of the centra dorsally elevated relative to the posterior faces. This is in contrast to the posterior cervical centra which have horizontally aligned faces. The ventral sides of the anterior centra are characterized by two anteroposteriorly-oriented paired fossae separated by a low keel (Figs.12, N, T, Y, EE, II, 13, F), as observed in the primitive nodosaurid *Animantarx* [97]. The dorsal ends of the neural spines are expanded transversely. AR-1-638/10 may either be the last cervical vertebra or the first dorsal vertebra based on the position of the parapophyses.

There are two complete cervical ribs preserved for the holotype. AR-1-450/10 is a relatively anterior cervical rib (Fig. 12 G, H) and AR-1-4452/10 is a posterior cervical rib. There is no evidence of fusion of cervical ribs to the cervical vertebrae as in the ankylosaurid *Saichania* [105], [106] or *Ankylosaurus* [107]. The cervical ribs are Y-shaped overall and much like the cervical ribs of other ankylosaurs such as *Silvisaurus* [76], [78], [82].

Several amphiplatan to amphicoelus dorsal vertebra are preserved: eight for the holotype AR-1/10 and nine for the paratype AR-1/31. The diapophyses originate at the level of the post-zygopophyses at the dorsal extent of the neural canal. The more anterior vertebrae have large cylindrical amphiplatan centra which lack a constricted ventral keel with circular neural canals and fused ribs (AR-1-448/10, 478, and 535). The broad transverse processes are T-shaped in cross-section and angled dorsally, unlike the laterally directed transverse processes in *Polacanthus* [10], [38]. Two dorsal vertebrae from the holotype appear to be pathological with the centra overgrown by about 0.5 cm of lumpy reactive bone (Figs. 14, G–K, W–BB). One of these pathologic vertebrae (AR-1-535/10) has fused ribs (Fig. 14 G–K) although the other (AR-1-430/10) does not (Fig. 14 W–BB). Two additional dorsal vertebrae (AR-1-478/10, 448) with fused ribs are not pathologic (Fig. 14 L–V). More posterior dorsal vertebrae have shorter, taller, more medially constricted centra, laterally compressed neural canals, more dorsally directed transverse processes, and lack fused ribs (AR-1-155/10, 322, and 556). The neural spines are thin and rectangular with narrowly expanded dorsal ends as in *Sauropelta* [99]. The neural spines are oriented dorsally as opposed to the posteriorly inclined neural spines of some other ankylosaurs such as *Sauropelta* [97]. None of the paratype vertebrae (AR- 1-3489/31, 3633, 3662, 3672, 3673, 3674, 3675, 3677 and 3704) have fused ribs (Fig. 15), suggesting that this character is ontogenetic because the paratype AR-1/31 represents a somewhat smaller (and presumably younger) individual than the holotype AR-1/10. More expanded neural spines are present in *Shamosaurus* [91].

There are a number of rib fragments preserved with AR-1/10, but there are only three (AR-1-331/10, 333, 476) relatively complete ribs (Fig. 16). As with most other ankylosaurs, the ribs are sharply arched and L-shaped in cross-section proximally in anterior ribs and broadly arched and T-shaped in cross-section proximally in more posterior ribs.

The sacrum is not preserved in AR-1/10 other than an anteriormost centrum (AR-1-154/10) of the synscacrum (Fig. 17 W, X). However, for the paratype, AR-1-3466/31, there is a largely complete but fragmented synsacrum (Fig. 17 A–V) that includes an interpreted anteriormost synsacral centrum (AR-1-3451/31), more of the anterior synsacrum composed of two dorsal centra (AR-1-3450/31), four sacral vertebrae with the sacral ribs from the left side (AR-1-3446/31), two sacral ribs from the right side (AR-1-3452/31, 3460), and one caudosacral vertebra (AR-1-3512/31). Given that at least one intermediate and one anterior fused synsacral dorsal vertebra are missing, the vertebral formula for the synsacrum would be five or more dorsosacral vertebrae, four sacral vertebrae, and one sacrocaudal vertebra. The entire synsacrum would have been over 50 cm long and measures about 44 cm across the sacral ribs. The middle section of the preserved dorsal synsacrum thins anteriorly from about 7 cm wide to about 5.5 cm wide. It then expands again anteriorly as indicated by the anteriormost centrum of the synsacrum. This differs from the sacrum of *Euoplocephalus* [108] and *Saichania* [106] in which each centrum making up the synsacrum is constricted medially. The sacrum is distinctive in being more strongly arched anteroposteriorly than other described ankylosaur sacra. The neural spines are dorsoventrally shorter than the height of the centra and are fused into a vertical sheet of bone along the length of the sacrum. The caudosacral neural spine is longer and unexpanded, transitional in form between the sacral neural spines and those of the proximal caudal vertebrae. The neural spines are broken off the anterior end of the synsacrum. The ventral side of the sacrum and anterior synsacrum is longitudinally depressed. The distal ends of the sacral ribs are expanded and the most robust medial sacral rib is about 50% taller (9.4 cm) than wide (6 cm) at its attachment with the ilium. There is no sign of expansion of the dorsal termination of the neural spine on the sacrocaudal vertebra. Additionally, the caudal rib is reduced compared to the sacral ribs.

The sacrum of *Struthiosaurus languedocensis* [31] is similar overall, but based on the description is not so strongly anteroposteriorly arched as in *Europelta*. Similarly, the sacrum of *Hungarosaurus*, as exhibited at the Hungarian Natural History Museum, appears to be moderately arched. The moderate angulation of the faces of the sacral centra (somewhat wedge-shaped in lateral view) in *Anoplosaurus* [17] indicates that a moderately arched sacram may have been present in this taxon as well. Among North American nodosaurids, we have observed only a moderate anteroposteriorly arching of the synsacrum of *Silvisaurus*, which appears to be restricted to the posterior part of the sacrum and two sacrocaudals. In other ankylosaurs, the downward flexure of the tail from the hips is taken up in the proximal caudal vertebrae as in *Mymoorapelta* [84], [109] and *Euoplocephalus* [70], [82].

Only three proximal caudal vertebrae (AR-1-562/10, 635, 636) are present (Fig. 18 A–F, J–O, V–AA). The proximal-most caudal vertebrae are not preserved for the holotype. The preserved vertebrae probably represent caudal vertebrae positions in the interval of about 3–7. The centra are anteroposteriorly shorter than dorsoventrally tall and somewhat wedge-shaped in anterior and posterior views. The posterior chevron facets are well developed. The neural spines are inclined posteriorly and the dorsal ends of the neural spines are only slightly expanded transversely as in *Gargoyleosaurus* [95] and some other ankylosaurs such as *Cedarpelta* [86], *Edmontonia* [110], *Hungarosaurus* [32] and *Euoplocephalus* [70], [82]. The neural spines are strongly expanded in most polacanthids such as *Mymoorapelta* [84], [109], *Gastonia* [83], and *Polacanthus* [10], and some North American nodosaurids such as *Sauropelta* [99], and *Silvisaurus* [76]. The neural spine of AR-1-562/10 is broken, erroneously giving it the appearance of being strongly inclined posteriorly. The caudal ribs (transverse processes) in *Europelta* originate high on the sides of the centrum and angle ventrally proximal to flexing laterally, giving them a dorsally concave profile in anterior view like *Hungarosaurus*, *Struthiosaurus*, and *Peloroplites*, and unlike the ventrally flexed caudal ribs of many polacanthids [10], [84], [109] and the caudal vertebra assigned to “*Acanthopholis*” [17] or straight caudal ribs of *Gargoyleosaurus* [95], *Cedarpelta*, *Peloroplites* [86], and *Edmontonia* [87]. The proximal caudal ribs of *Hylaeosaurus* differ in being swept back posteriorly [111]. The lateral terminations of the caudal ribs do not expand dorsoventrally as they do in *Peloroplites* [86] and *Struthiosaurus*, which actually appear to bifurcate [25], [26].

Additionally, there are four chevrons preserved from about the same region of the tail (AR-1-560/10, 561, 569, and 4451) of which three are illustrated (Fig. 18 G–I, P–U). The proximal chevrons are approximately as long as the neural spines as in most other ankylosaurs. They are relatively straight and expanded into teardrop shapes distally in lateral view. Unlike in many ankylosaurs, there is no fusion of proximal chevrons to their respective caudal vertebrae as in *Pinacosaurus* and *Saichania* [105], [106], *Ankylosaurus* [107], [112], and *Edmontonia* (ROM 1215) [87].

Several more distal caudal vertebrae are preserved in the paratype. The two most proximal of these (AR-1-3348/31, AR-1-3717/31) have centra of nearly equal height, width, and length, with a ventral groove, and caudal ribs shorter than the diameter of the centrum that extend laterally and angle posteriorly (Fig. 19 A–J). The chevron facets are well developed with the posterior facets more strongly developed than the anterior facets. The neural spines are not developed and the zygapophyeses only extend a short distance beyond the anterior and posterior margins of the centra. These vertebrae are interpreted to represent mid-caudal vertebrae. Two more distal mid-caudal vertebrae (AR-1-3616/31, AR-1-3716/31) are similar in morphology except that the caudal ribs are reduced to anteroposteriorly directed ridges on the lateral margins of the centra (Fig. 19 K–N). Their neural spines incline posteriorly, merging with the postzygapophyses as posterior processes extending laterally past the faces of the centra to overlie and articulate between the paired prezygapophyses of the immediatly distal vertebra. This morphology is retained in the distal caudal vertebra. More distally, as in AR-1- 2950/31, 3206, 3243, 3265, 3478, and 3615, the caudal ribs are lost and the centra become more elongate (Fig. 19 O–FF). Unlike many ankylosaurs, the faces of the centra maintain a well-rounded to heart-shaped surface distally down the caudal series [82]. For many of these vertebrae, ventrally anteroposteriorly elongated skid-shaped (inverted T) chevrons are fused to the posterior chevron facets. Fusion of distal chevrons to their respective vertebrae is widespread among ankylosaurs [84], [106], [110] although it is not present in some, such as *Nodosaurus* [113]. One pair of distal caudal vertebrae is fused by their mutually shared chevron (Fig. 19 GG–II) such as has been documented in *Mymoorapelta* [84]. The most distal four caudal vertebrae (Fig. 19 JJ–LL) and their chevrons are fused together in AR-1-3204/31 to form a tapering, terminal rod of bone at the end of the tail somewhat similar to that of *Sauropelta* [71].

### Pectoral Girdle

Parts of the right scapulocoracoid are preserved. A portion of the distal scapular blade (AR-1-429/10) is preserved with a portion of the distal ventral margin missing with a curved section broken away. There is no evidence of any distal expansion of the scapular blade as in many nodosaurids [94].

The coracoid (AR-1-657/10) is preserved with only the most proximal portion of the scapula fused on (Fig. 20 D–H). It appears to have been sheared off just dorsal to the suture between the coracoid and the scapula, perhaps in the process of removing the overlying coal seam. The coracoid is relatively equidimensional (201.3 mm long by 186.5 mm tall) relative to the elongate coracoids characteristic of many other nodosaurids [114] such as *Peleroplites* [86], *Texasites* [77], [115], and *Animantarx* [97]. The medial surface is concave and the lateral surface is convex giving it a bowl-shaped appearance. The ventral margin is evenly convex as in many polacanthids and nodosaurids and there is no anteroventral process as in all ankylosaurids, including *Shamosaurus* [91], [94]. The articular surface of the ventrally directed glenoid is wide, bounded by a flange that extends beyond the medial surface of the coracoid.

Both xiphisternal plates are preserved (Figure 20I–L). The best preserved xiphisternal is approximately 350 mm long. They appear to be arcuate flat bones. Xiphisternal plates are only known in a few nodosaurids, but those of *Europelta*, whereas similar in overall shape to other nodosaurid xiphisterna, are not fenestrate or scalloped along their margins as in North American nodosaurids for which they are known [82], [87], [116].

### Forelimb

Parts of both humeri are preserved. The right humerus (AR-1-655/10) is represented by the proximal end (Fig. 21 A–D). It is 249.2 mm wide with a well-developed proximal head 91.9 mm wide that extends onto the posterior side of the humerus. Distinct notches separate both the laterally directed deltopectoral crest as in nodosaurids such as *Sauropelta* [70], [71], [99] and the internal tuberosity from the humeral head. The deltopectoral crest extends lateraly from the humerus and is not flexed anteriorly as in polacanthids and ankylosaurids [94].

The left humerus (AR-1-327/10) is represented by a midshaft for which both the proximal and distal ends appear to have rotted off and the core of the shaft has rotted away (Fig. 21 E–H). The shaft is deeply waisted relative to the proximal and distal ends. Although relatively uninformative, enough of this humerus is preserved to indicate that the deltopectoral crest would have made up less than 50% of the length of the humerus as in nodosaurids [71], [117] and in the basal ankylosaur *Mymoorapelta* (Kirkland, pers. obs.) compared to the longer deltopectoral crests of ankylosaurids [70], [71]. Overall, the humerus of *Europelta* is similar in proportions to *Niobrarasaurus* [118], [119]. The wide proximal end of the humerus figured by Ősi and Prondvai [120] as cf. *Struthiosaurus* is similar to that of *Europelta*, whereas the humerus of co-occuring *Hungarosaurusis* is more slender proportionally.

Among the nine unguals preserved for AR-1/31, one specimen (AR-1-3711/31) may represent a manual ungual. It is more equidimensuional than the other eight more elongate unguals.

### Pelvic Girdle

The right ilium of AR-1/10 is fused with its ischium and pubis (AR-1-479/10) which are flexed medially due to compaction (Fig. 22 A–D). The acetabulum is completely enclosed as in all derived ankylosaurs [70], [71], [82], [94], [108]. Only *Mymoorapelta* is known to retain an open acetabulum [84], [109]. The acetabulum is directed verntrally and is situated medially near the contact of the ilium with the sacrum so that the ilium extends far out beyond the acetabulum laterally for a distance nearly equal to its width. The lateral and anterior margins of the laterally oriented ilium are broken away. The prepubic portion of the ilium diverges from the midline of the sacrum at about 30 degrees and is thickened ventrally along its midline. Large, fairly equi-dimensional, closely appressed osteoderms (7-10 cm in diameter) cover the dorsal surface of the ilium posterior to and medial to the acetabulum. As discussed below, this morphology of sacral armor compares well with “Category 3” pelvic armor of Arbour and others [121]. Anteriorly, the smooth dorsal surface of the ilium is exposed. The pubis is fully fused to the anterior margin of the ischium with no visible sutures; its presence is indicated by a slot-shaped foramen along the anterior side of the ischium. This foramen represents the obturator notch between the postpubic process and the main body of the pubis as in *Scelidosaurus* and stegosaurs [122]. The distal end of the ischium is broken away. Additionally, AR-1-129/10 is a poorly preserved, proximal left ischium with the pubis fully fused to its anterior margin (Fig. 22 E, F).

Beyond some relatively uninformative fragments of the ilium (Fig. 23 A–C), AR-1/31 includes both the right (AR-1-3648/31) and the left (AR-1-3649/31) ischia with fully fused pubes (Fig. 23 D–M). Both exhibit the slot-shaped foramen along the anterior side of the ischium formed by the obturator notch. The proximal ends appear enrolled such that the anterior and posterior margins are nearly parallel due to compaction. Both display an anterior kink at their distal end as in *Cedarpelta* [86], [88], but overall are straight-shafted as in the Ankylosauridae [70], [82], [123] and the other European nodosaurids *Struthiosaurus* [31] and *Hungarosaurus* [32]. The distal end of the left ischium is the best preserved and measures 299.9 mm long along its anterior margin, including the fully fused pubis forming an ischiopubis. Given the asymmetry of the proximal end of the fused ischium and pubis and the position of the obturator foramen, it appears that the pubis still makes up some of the acetabular margin. The contact between the ilium and the fused ischiopubis is straight with about one-fourth to one-third of the acetabulum formed by the fused ischiopubis.

A straight ischium has been considered to be the primitive character state for ankylosaurs, with the bent ischium of *Polacanthus* and nodosaurids, a derived character [63], [82], [83], [94], [114], [123]. It is possible that as opposed to being primitive, a straight ischium may be secondarily acquired in the ankylosaurids and European nodosaurids. The only known ischium from the Jurassic ankylosaur (*Mymoorapelta)* is bent, a trait that is also observed in some stegosaurs such as *Kentrosaurus* [124]. Stegosaur ischia, even when straight, have an angular thickening near the mid-point of the posterior margin [124] that is shared by the polacanthids *Mymoorapelta* (Kirkland pers. obs.) and *Gastonia* [83]. *Europelta* is the oldest known ankylosaur preserving a straight ischium. The slight kink in the distal end of the ischium of *Europelta* suggests the straight ischium in European nodosaurids and ankylosaurids is achieved by shortening the ischium distal to the bend.

### Hindlimb

The right femur, tibia, and fibula were closely associated (Fig. 24 A–F). The robust right femur (AR-1-3244/31) is 502.9 mm long and 178.9 mm wide at the proximal end and has been flattened anteroposteriorly, with the most distortion to the mid-shaft region. The femoral head is distinct with much of its articular surface directed dorsally and only somewhat medially. It forms an angle of about 115° with the long axis of the femur. The femoral head is directed more dorsally under the ilium in polacanthids [7], [12], [82], [95], [125], and several nodosaururids. In addition, the femoral head of *Europelta* is expanded such that it overhangs the femoral shaft both anteriorly and posteriorly. The greater trochanter is well demarcated from the femoral head by a constriction across the proximal end of the femur, and the anterior trochanter forms a ridge ventral to the greater trochanter that is fully fused to the femur. The robust fourth trochanter overlaps the midpoint of the femoral shaft and its midpoint is located proximal at the midpoint of the femur. Polacanthids and nodosaurid ankylosaurs have this configuration, whereas in ankylosaurids the fourth trochanter is distal to the middle of the shaft [63], [82], [95], [120], [125]. The distal end of the femur is flattened and forms a planar articular surface relative to the straight femoral shaft. The intercondylar notch is not expressed ventrally, and is better developed posteriorly than anteriorly

The right tibia (AR-1-3237/31) and fibula (AR-1-3238/31) were closely associated (Fig. 6) and post-depositionally compressed. Compression has distorted the distal end of the tibia such that the wide posterior surface is twisted counterclockwise in line with the wide lateral side of the anterior end relative to the orientation of the proximal and distal ends of the tibia in most other ankylosaurs, such as *Mymoorapelta* [84] (Kirkland, pers. obs.). The fibula was taphonomically displaced ventrally and with the ventral end rotated posteriorly relative to its position in life with the tibia.

The tibia is 458.8 mm long and robust for its entire length (Fig. 24 G–K, Q) as in *Cedarpelta* [86]. The proximal end is 169.2 mm wide by 93.1 mm wide and its distal end is 146.8 mm wide by 70.2 mm. It is significantly more narrowly waisted in *Mymoorapelta* [84], *Gastonia* [83], *Polacanthus* [7], [12], [18], *Sauropelta* [69], [71], [99], [108], *Peloroplites* [86], and in *Zhejiangosaurus* [126] and ankylosaurids like *Saichania* [106]. The cnemial crest is broadly rounded. The even curvature of the distal end of the tibia suggests that the astragalus was fully fused to it with no evident sutural contact as in most ankylosaurs [63], [82], [121]. The astragalus is not fused to the distal end of the tibia in *Mymoorapelta* [84], *Gastonia* [83], *Hylaeosaurus* [11], and *Peloroplites* [86].

Generally, ankylosaurids have tibiae that are less than two-thirds the length of their femora, as opposed to nodosaurids which have proportionally longer lower leg elements [127]. With a tibia to femur ratio of 0.91, *Europelta* has the proportionally longest tibia of any ankylosaur for which this ratio is known. Both *Cedarpelta* and *Peloroplites* have relatively longer tibiae than other ankylosaurs [86], with a tibia to femur ratio of 0.82 in both. *Peloroplites* differs in its proportionally more narrowly waisted tibial shaft.

The fibula is 395.5 mm long (Fig. 24 L–P, R) and laterally flattened. The proximal end is not expanded anteroposteriorly, such that the slender fibula changes little in size and shape from the proximal to distal end. In lateral view, the proximal end is rounded and the distal end is concave. In cross-section, it is flattened medially and convex laterally. It is longer relative to the tibia than in most other ankylosaurs [108].

A calcaneum (AR-1-3289/31) was identified in association with the lower right leg of AR-1/31. It is laterally compressed, convex laterally and concave medially (Fig. 24 S, T). Its dorsal margin is flattened where it would articulate with the fibula. Calcanea are practically unknown in ankylosaurs, but one has been identified in the juvenile specimen of the derived ankylosaur *Anodontosaurus* [128]. The type of *Niobrarasaurus coleii* preserves an articulated lower hind limb, with an astragalus fully fused with the tibia and possessing an articulation with the distal end of the fibula and an unfused calcaneum of similar morphology to that of *Europelta* [118]. The calcaneum is fully fused to the distal end of the fibula in *Saichania* [106].

A number of metatarsals and phalanges are associated with AR-1/31. The metatarsals have subrectangular proximal ends, indicating that they were closely articulated in a well-integrated pes in life (Fig. 25 A–W). The pedal phalanges (Fig. 25 X–JJJ) are short, as in other ankylosaurs. There are eight relatively large, elongate, spade-like unguals (Fig. 25 KKK–WWWW) of a morphology similar to pedal unguals in other ankylosaurs in which the unguals are nearly as long as the digits[82], which indicates that portions of both feet are present in AR-1/31. We interpret that the pes of *Europelta* possesses four pedal phalanges as in most other nodosaurids [80]. *Liaoningosaurus* has three digits on the pes. The eight similar unguals are interpreted as pedal unguals and the smallest ungual (Fig. 25 XXXX–BBBBB) is interpreted as an isolated manual ungual. The overall proportions of the preserved pedal elements are similar to those of *Niobrarasaurus* [119], which also has pedal unguals nearly as large as its metatarsals.

### Armor

There was an abundance of dermal armor recovered with both AR-1/10 and AR-1/31. On comparison with the quarry maps, none of the osteoderms appears to be preserved in situ with any of the skeletal elements or with each other, and there is no fusion between any of the osteoderms recovered. Therefore, the armor has been divided into several broad morphotypes for the purpose of description and comparison to armor described for other ankylosaurs. Although morphotypes and terminologies have been proposed [129], [130], no system fits for all armor types in all ankylosaurs. A number of researchers have divided armor into types as in Type 1, 2, etc. [131]; for this discussion the armor types are alphabetized to ensure minimal confusion with previous descriptions. The term osteoderm is used to describe relatively larger dorsal and lateral armor elements with the presence of an external keel or tubercle, whereas the term ossicle describes relatively smaller dermal armor lacking a keel, in the sense of Blows [130]. It is recognized that a consistent methodology for describing armor is achievable, but must be done within a phylogenetic framework to be of maximum utility.

Osteoderm surface texture may be broadly useful in differentiating ankylosaurids from nodosaurids [132], [133]. The vast majority of the osteoderms examined in *Europelta* has a moderately rugose texture with sparse pitting more in keeping with nodosaurids and basal ankylosaurids rather than more derived ankylosaurids. Whereas histological studies have proven useful in the study of thyreophorans [132], [134], [135], that is beyond the scope of this study.

It is noteworthy that no portions of distinct cervical rings were recovered, although cervical vertebrae are known for both skeletons of *Europelta*. Additionally, only one spine from the cervical or pectoral region was tentatively identified. We postulate that these elements were lost through the process of coal removal or may have been taphonomically removed from the skeletal associations. Only the discovery of additional specimens of *Europelta* can further reveal the presence of cervical half-rings.

#### Type A armor

An isolated fragmentary spine (AR-1-128/10), possibly from the cervical or pectoral region, is recognized from the holotype (Fig. 26 A–D). It appears to represent only the anterior half and may have been cut in two as the overlying coal was removed. This sharp, broken margin reveals an asymmetric, Y-shaped cross-section. The base flares more and is is less excavated than in a Type 2 caudal plate, suggesting that it was positioned on a broad flank of the body. From the possible anterior margin, the spine slopes posteriorly 15 cm to the broken margin in a gradual arc. There is no indication that the spine could not have been longer. The spine is compressed as in the cervical spines of *Sauropelta* [77], [99] and *Edmontonia* [110], [136], and the pectoral spines of *Gastonia* [83] and *Polacanthus* [7], [10]. The base is asymmetrical in a manner similar to the elongate osteoderms in *Mymoorapelta* [84], with one side of the base extending lower anteriorly and the other posteriorly. There is no evidence of a basal plate incorporated into fusion of the cervical half-ring as in mature ankylosaurs like *Mymoorapelta* [84] *Gargoyleosaurus* [85], [95], *Gastonia* [83], *Polacanthus* [10], [130], and *Sauropelta* [77], [99]. This may relate to the anchoring of larger elements into the dermis in *Gastonia* and *Polacanthus* [130]. We tentatively interpret AR-1-128/10 as a pectoral spine. However, if the complete element extends beyond the break for more than twice the length of the preserved portion, it would fall into the category of Type B armor, although that is unlikely because it is more massive form than the Type B elements.

#### Type B armor

Dorsoventrally compressed, hollow, asymmetric-based plate-like osteoderms with sharp anterior and posterior edges and lateroposteriorly directed apices are identified for AR-1/10 (Fig 26 E–J) and AR-1/31 (Fig. 27 A–L). Similar large osteoderms have been described as caudal plate ostederms in *Mymoorapelta* [84], [109], *Gargoyleosaurus* [85], [95], *Gastonia* [83], and *Polacanthus* [8]-[10], [38], [130]. Similar, more anterorposteriorly symmetrical caudal plate osteoderms are also known in *Minmi* [137], [138] and several Asian ankylosaurids [131]. The few plate-like osteoderms of this morphology that are identified in *Europelta* are mediolaterally shorter and anteroposteriorly longer with a more posteriorly swept apices. Two pairs of similar plates are known for the holotype of *Sauropelta* (AMNH 3032), with one of the larger plates being illustrated [99]. One plate from the Yale collections of *Sauropelta* has a unique double apex (YPM 5490). Given the rarity of Type B armor in *Sauropelta* and *Europelta* we hypothesize that caudal plates in these nodosaurids ran down the sides of the tail but decreased in size more rapidly, such that long-keeled osteoderms of Type E morphology made up the lateral armor down most of the length of the tail. It is also possible that these large plate-like osteoderms were on the lateral margin of the sacrum as has been documented by Carpenter and others [106] in *Saichania*. *Struthiosaurus* preserves several osteoderms of this morphology that have been reconstructed as in *Polacanthus* as being medial, dorsally-projecting caudal osteoderms [25], [26]. The relative rarity of these plate-like osteoderms suggests that they were restricted to the base of the tail as well.

#### Type C armor

Both AR-1/10 (Fig. 28 A–H, O, P) and AR-1/31 (Fig. 29 A– F) preserve fairly large (∼15–25 cm long) subrectangular to subtrapezoidal, solid osteoderms with low, evenly developed keels running down the long axis of the osteoderm either medially or to one side of the mid-line. Their distal and medial surfaces are subparallel and the entire plate may be slightly flexed across the short axis perpendicular to the crest. The straight, longer margins of these plates appear to have been tightly affixed but not fused to adjoining osteoderms. Armor of Type C morphology is not common but is most similar to medial cervical osteoderms of half-rings, and most distinctively, across the mid-line of the pectoral region in some nodosaurids such as *Stegopelta* [138], *Niobrarasaurus* [140], [141], *Panoplosaurus* [74], [101], and *Edmontonia* [74], [110].

#### Type D armor

Both AR-1/10 and AR-1/31 preserve large (∼10-20 cm long) asymmetric, diamond (Fig. 28 I–N, Q–T; Fig. 29 M–P) to tear-drop shaped (Fig. 29 G–L, Q, R, U,V) osteoderms with a long keel rising to an apex medially to posteriorly and in some specimens extending past the posterior margin of the base. They are distinguished from Type E osteoderms because they are wider than 50% of their length. The wider osteoderms are thinner and more solid than the narrower osteoderms with small pockets under the apices. The more diamond-shaped forms may be more closely appressed to each other in anterior bands similar to Type C armor.

Type D Armor is widely known in the nodosaurids such as *Sauropelta* [99], *Panoplosaurus* [101], and *Edmontonia*. *Gastonia* is documented to have similar armor [142], although more solid in cross section with less basal excavation, which occurs in oblique rows anterior to the sacrum with each osteoderm separated by a single row of small Type H ossicles. This pattern is similar to the dorsal dermal ornamentation documented for the ankylosaur *Tarchia* by Arbour and others [130], except that in *Tarchia* most of the intermediate scales lacked ossified cores. Similar armor is known from the lateral sides of the legs in some ankylosaurs such as *Saichania* [106].

#### Type E armor

Both AR-1/10 and AR-1/31 preserve large (10-15 cm long) moderately asymmetric osteoderms more than twice as long as wide with a long keel higher on the assumed posterior end (Fig. 28 Q, R, II–NN; Fig. 30 G–FF). These osteoderms have proportionally more deeply excavated bases than Type D armor, have chevron-shaped cross-sections, and are distinguished from Type D armor by their width being less than 50% of the length. Type E armor is gradational with Type D armor (Fig. 28 S–T; Fig 29 A–F) and may represent lateral or distal armor from the trunk of the body and along the sides of the tail. This armor type is present in *Sauropelta* [99] and *Texasetes* [115]. Similar armor is present on the sides of the limbs in *Scelidosurus* and *Saichania* [106].

#### Type F armor

Medium to large (∼5-15 cm long) oval to circular osteoderms of low profile with a median keel extending into an apex near or overhanging the posterior margin of the osteoderm are represented in both AR-1/10 (Fig. 28 U–Z) and AR-1/31(Fig. 29 W–VVV). The basal surface of the osteoderm is generally solid except for a small pocket under the apex, reminiscent of Type D armor. Less commonly, the base may be more extensively excavated. Armor of this morphology is abundant in many nodosaurids and makes up the major elements of the armor of *Sauropelta* anterior to the sacrum in AMNH 3036 [142] and is present in *Panoplosaurus* [101]. These osteoderms may reside within more expansive spaces among the larger dorsal armor as in *Edmontonia* (AMNH, 5665) and the polacanthids [81], [82], [93], [107], or may be major armor elements on the posterior portion of the sacrum as in *Sauropelta* (AMNH 3036). They may also lie on the tail between the Type B caudal plate-like osteoderms, or could be arranged along the lateral side of the limbs as in *Saichania* [106].

#### Type G armor

One piece (AR-1-192/10) of flat, oval to subtriangular armor (AR-1-192/10) from AR-1/10 is about 12 cm long and 7 cm wide and is about 0.5 cm thick throughout (Fig. 28 AA, BB). A pair of similar, osteoderms from the *Sauropelta* specimen AMNH 3032 was curated with a note from the collector, Barnum Brown, stating that these distinct osteoderms were associated with the forelimbs. Therefore, we suggest a similar position for Type G armor in *Europelta*.

#### Type H armor

Small (∼1-4 cm long) solid ossicles are abundant, with 71 examples from both AR-1/10 (Fig. 31) and AR-1/31 (Fig. 32) illustrated. These ossicles range in shape from round, to oval and even irregularly shaped, and are probably filling in the spaces between larger osteoderms. Small interstitial ossicles are not known for every ankylosaur taxon, but appear to be present in many nodosaurid taxa such as *Sauropelta* [99], [143] and E*dmontonia* [74], [136], in polacanthid ankylosaurs such as *Gastonia* [83] and in some ankylosaurids such as *Tarchia* [131], in which epidermal scales interstitial to osteoderms do not preserve deeper, interstitial ossicles. Their absence may be real, in that they never form deep to the epidermal scales, taphonomic, in that they are selectively transported away because of their small size and low density, or ontogenetic; in that they only ossify late in ontogeny. The surface texture of *Gastonia* ossicles is smoother than those of *Europelta*.

#### Sacral armor

Armor is present on the posterior margin of the ilium AR-1-479/10. It is composed of large, subequal-sized (7-10 cm) osteoderms that are tightly sutured together (Fig. 22 A) as in the poorly known *Stegopelta* [139], *Nodosaurus* [113], *Aletopelta* [127], and *Glyptodontopelta* [132], [144]. These low-relief ossicles lack a central apex or keel. The boundary between the margins of the osteoderms and the area devoid of osteoderms on the ilium is sharply demarcated along the margins of unbroken osteoderms, suggesting the armor was not coossified as in *Aletopelta* [127] and unlike the fully fused sacral armor in the polacanthids *Polacanthus* and *Gastonia* [63], [83]. This form of pelvic armor fits that of Arbour and others' Category 3 pelvic armor [121].

Additionally, there is a unique osteoderm AR-1-653/10 that has a large, posteriorly-curved, plate-like keel extending out from the surface that, considered in isolation, is comparable in size and morphology to Type B armor (Fig. 26 K–N). The base is smooth and gently convex, suggesting it may have been closely appressed to the more anterior portion of the ilium. In overall morphology, this large osteoderm is comparable to the spine-bearing armor plate-like osteoderm identified in *Hungarosaurus* and interpreted to be present in *Struthiosaurus* [33].

#### Unique armor pieces

Some irregularly shaped armor specimens are not represented by more than one element among this material or in the armor from other taxa. At this time, we can offer no positional interpretation of this armor. AR-1-447/10 is an irregular mass of what we interpret as an osteoderm, although it could be sacral armor (Figure 28 CC–FF). AR-1-438/10 is a small, cap-shaped shaped with a small excavation in the center of the external surface (Fig. 28 OO, PP). Two small, deeply basally excavated, oval osteoderms (Fig. 30 GG–JJ) were collected from AR-1/31(AR-1-3239/31, 3721). These osteoderms lack the external excavation.

## Discussion

*Europelta* (Fig. 33) can be distinguished from any of the ankylosaurs assigned to the Polacanthidae (sensu Kirkland's Polacanthinae [83] and Carpenter's Polacanthidae [63] from the Upper Jurassic and Lower Cretaceous as defined by Yang and others [64]; see Terminology) by its rounded, tear-drop shaped skull and a suborbital horn developed on the posterior portion of the jugal and the quadratojugal posterior to the orbit, as opposed to a triangular-shaped skull that is widest at the posterior margin and a suborbital horn developed exclusively on the jugal (as seen in polacanthids). Post-cranially, it can also be distinguished from polacanthids, by its elongate lower hind limbs, the apparent rarity of cervical, pectoral, and thoracic spines, and reduction in the number of caudal plate-like osteoderms. Likewise, it has an abundance of Type D, asymmetric, tear-drop shaped osteoderms like those observed in many nodosaurids and absent in all polacanthids.

*Europelta* is also distinguished from derived ankylosaurids by its weakly ornamented teardrop-shaped skull in which the lower temporal opening is visible in lateral view. The absence of a tail club also distinguishes the taxon from these ankylosaurids. More basal “shamosaurine grade” ankylosaurids [63], [86] are more similar to *Europelta*, but also have the lower temporal openings completely obscured laterally by expanding the lateral margin of their skulls. “Shamosaurine grade” ankylosaurids also possess skulls that are approximately as wide mediolaterally between the orbits as they are across the posterior margin.

*Europelta* shares a number of derived characters with nodosaurids [71], [72], [83], [94], [114]. It has a tear-drop shaped skull that is longer than wide with its greatest width dorsal to the orbits, whereas the short, boxy skulls of *Minmi* and all anklosaurids are essentially as wide at the posterior edge of the skull, as are the elongate skulls of “shamosaurine-grade” ankylosaurids. Grooves in the remodeled textured skull roof define epidermal scale impressions, with the largest covering the frontoparietal area. Although poorly preserved, the laterally extensive pterygoids are pressed up against the anterior face of the braincase. All known nodosaurid scapulae have a prominent acromion process extending on to the blade of the scapula that terminates in an expanded knob. Unfortunately, this portion of the scapula is as yet unknown in *Europelta*.

Some character states considered typical of nodosaurids are absent in *Europelta*. Instead of having a distinct hourglass-shaped palate typical of nodosaurids [70], [71], [82], [83], [114], the upper tooth rows show less lateral emargination and diverge posteriorly. This is also true of *Silvisaurus*, which also shares an expanded lateral wall of the skull [76], [77]. The coracoid of *Europelta* is nearly as long as it is tall, whereas in other nodosaurids, for which the corocoid is known, it is expanded anteriorly and longer than tall [71], [72], [83], [94], [114].

The only other Early Cretaceous nodosaurid to have large cranial scales as in *Europelta* is *Propanoplosaurus*, known only from an embryonic to hatchling specimen from the base of the Potomac Group of Maryland [145]. However, only the anterior cranial scales are well defined in *Propanoplosaurus*, whereas only the posterior scale pattern in *Europelta*. The unusual preservation and extremely small size of *Propanoplosaurus* lead us to suspect that the fossil preserves the actual scales overlying the skull and not the remodeled skull roof, because this is such a young specimen and remodeling of the cranial bones is not expected to have occurred so early in ontogeny [129], [146].

Additionally, a number of important characters traditionally used to define nodosaurids are not known in *Europelta*, as yet, because of the missing anteroventral half of the scapula and the absence of premaxilla and surangulars. Thus, the presence absence of premaxillary teeth, if the tooth row joined the margin of premaxillary beak, the morphology of the naris, the height of the coronoid process, and the morphology of the acromion process are unknown for *Europelta*.

*Europelta* is distinguishable from European nodosaurids from the Albian through the Cenomanian. The juvenile *Anoplosaurus* from the Albian Gault Clays of southern England differs in a number of characters, such as possessing a proportionally longer coracoid, a narrower proximal end of the humerus, and a femur with a separate anterior trochanter [17] although the latter two characters are consistent with the juvenile nature of *Anoplosaurus*. No pectoral spines of the morphology described for “*Acanthopholis*” from the Cenomanian Lower Chalk in southern England by Huxley [13] are known in *Europelta*. Additionally, the tall teeth assigned to “*Acanthopholis*” are distinct in the long apicobasal ridges extending from the denticles to the root on medial and lateral faces of the teeth, and in the presence of caudal ribs that extend laterally and flex ventrally, whereas the caudal ribs in *Europelta* extend ventrolaterally and flex laterally [16], [17]. *Europelta* is like other Late Cretaceous European nodosaurids in having a short symphysis for the predentary, a mediolaterally wide and anteroposteriorly thin quadrate, an anteroposteriorly arched sacrum, and a straight ischium [21], [32].

The domed skull and elongate cervical vertebrae in *Struthiosaurus* clearly distinguish it from *Europelta*. Likewise, *Hungarosaurus* also has more elongate cervical vertebrae [32]. Both *Hungarosaurus* and *Struthiosaurus* possess a pair of spines on the anterior portion of the pelvis [33], whereas we interpret the presence of a pair of upright plate-like armor elements in this position in *Europelta* (Fig. 33).

The lateral wall of the skull in most North American nodosaurids is typically narrow [82], whereas in *Europelta* it is relatively wider, although a broad notch along its posterior margin permits the caudal margin of the lower temporal opening to be observed in lateral view. This morphology in *Europelta* is similar to that in the nodosaurids *Silvisaurus* [76], [77] and *Peloroplites* [86]. Although, the skull of *Struthiosaurus transylvanicus* is highly reconstructed [22], it appears that the lateral wall of the skull is expanded laterally, whereas not completely obscuring the lower temporal opening. This character state is not known in other species of *Struthiosaurus*, but appears to be moderately developed in *Hungarosaurus* [32].

Comparisons of *Europelta* with the Asian”nodosaurids” *Zhongyuansaurus* [93] and *Zhejiangosaurus* [126] from the lower Upper Cretaceous of China hinges partially on the question of whether those taxa have been validly referred to Nodosauridae. Carpenter and others [86] noted that the skull of *Zhongyuansaurus* is morphologically similar to that of a “shamosaurine-grade” (like *Shamosaurus* and *Gobisaurus*) ankylosaurids and was the first shamosaurine-grade ankylosaurid documented to not have a tail club. However, its distal tail is modified into a stiffened structure of the same morphology as the “handle” of the tail club in more derived ankylosaurids [147], [148]. *Zhejiangosaurus* was assigned to the nodosaurids based on characteristics of the femur and sacrum, together with the lack of a tail club [126]. We hypothesize that it lacked a knob as in basal ankylosaurids, polacanthids and nodosaurids because ankylosaurids with a full tail club have distal free caudal vertebrae bearing caudal ribs at the base of the handle. Most of the distal caudal vertebrae of *Zhejiangosaurus* have raised ridges on the sides of the centra as in the distal vertebrae of polacanthids and nodosaurids. Additionally, whereas the position of its most proximal preserved caudal vertebrae is not known, morphologically, they do not appear to represent the most proximal caudal vertebrae. Thus, while *Zhejiangosaurus*' 13 preserved caudal vertebra are more than the number of free caudals preserved in most ankylosaurs with tail clubs (10 in *Saichania* [106] and *Dyoplosaurus* [148]), the total number of free caudals in its tail would appear to be more than the 14 in *Tarchia* [130] and 15 in *Pinacosaurus* [129]. Unlike nodosaurids, *Zhejiangosaurus* has an exceedingly low ratio of femur to tibia length of 0.46 similar to that of with ankylosaurids and polacanthids rather than nodosaurids. *Dongyangopelta* [149] was described as a second nodosaurid from the same area and stratum as *Zhejiangosaurus*, which was found to be its sister taxon in their phylogeny [149]. With few overlapping elements, we feel that the proposed differences between these taxa may be due to preservation, individual variation, or ontogeny. Additionally, given the presence of a pelvic shield and numerous caudal plate-like osteoderms in *Dongyangopelta*, we suggest that both specimens may pertain to the same taxon and represent the first polacanthid described from Asia. Given the recent description of the polacanthid *Taohelong* from the upper portion of the Lower Cretaceous of Gansus Province in western China [64], this hypothesis has added support. We also do not think that the partial ankylosaur skull reported from the lower Upper Cretaceous of Hokkaido, Japan [150] can be diagnosed as a nodosaurid with any confidence at this time, due to the incomplete nature of the specimen. Thus, we do not presently recongnize the presence of true nodosaurids in Asia.

In his seminal paper defining a bipartite division of the Ankylosauria into Ankylosauridae and Nodosauridae, Coombs [71] hypothesized that *Acanthopholis* (as a *nomen dubium* in which he would have included *Anoplosaurus*) and *Struthiosaurus* might represent a separate lineage of European nodosaurids. Unlike *Hylaeosaurus* (in which he included *Polacanthus*), these taxa had a well-developed supraspinus fossa developed anteriorly on the scapula as did all North American nodosaurids. This European lineage was hypothesized based on their small body size, presence of premaxillary teeth, and their possessing an unfused scapula and corocoid. Although, none of the characters are valid in defining such a group, our research on *Europelta* has resulted in supporting the taxonomic hypothesis of Coombs [71], [72] as correct, just for the wrong reasons.

### Relationships to Other Taxa

We use Struthiosaurinae to define the clade of European nodosaurs. Nopcsa [25] proposed Acanthopholidae as a family of relatively lightly built thyreophorans, that included *Acanthopholis* ( =  *Anoplosaurus*), *Polacanthus*, *Stegopelta*, *Stegoceras*, and *Struthiosaurus*. In 1923, he divided the Acanthopholidae into an Acanthopholinae and a Struthiosaurinae without comment [69]. Subsequently, he relegated the Acanthopholidae to a subfamily of the Nodosauridae, in which he also included *Ankylosaurus* and restricted the Acanthopholinae to *Acanthopholis, Hylaeosaurus, Rhodanosaurus, Struthiosaurus, Troodon* [26], [151]. This artificial grouping included a polacanthid ankylosaur [72], [83], a pachycephalosaur [152] and *Acanthopholis*, now considered a *nomen dubium* [17], [82]. Thus, the term Acanthopholinae is not acceptable for this newly recognized clade of nodosaurids. Thus, Struthiosaurinae is the next published term available to use for this clade and is derived from the first described and youngest member of this clade. Struthiosaurinae is defined as the most inclusive clade containing *Europelta* but not *Cedarpelta*, *Peloroplites*, *Sauropelta* or *Edmontonia*.

In order to determine the systematic position of *Europelta*, it was found that previous cladistic analyses [71], [72], [82], [83], [114], did not include many of the character states that we identify as significant in our research on Upper Jurassic and Lower Cretaceous ankylosaurs. A major weakness of these analyses is the limited recognition of postcranial skeletal and dermal characters that restricts the testing the phylogenetic relationships for taxa for which skulls are either poorly known or not known at all.

We present a character based definition of Struthiosaurinae as: nodosaurid ankylosaurs that share a combination of characters including: narrow predentary; a nearly horizontal, unfused quadrate that is oriented less than 30° from the skull roof, and mandibular condyles that are 3 times transversely wider than long; premaxillary teeth and dentary teeth that are near the predentary symphysis; dorsally arched sacrum; an acromion process dorsal to midpoint of the scapula-coracoid suture; straight ischium, with a straight dorsal margin; relatively long slender limbs; a sacral shield of armor; and erect pelvic osteoderms with flat bases. This suite of characters unites *Europelta* with the European nodosaurids *Anoplosaurus*, *Hungarosaurus* and all species assigned to *Struthiosaurus*. This clade of European nodosaurids has not been previously recognized. *Europelta* represents the earliest member of the European clade Struthiosaurinae.

### Biogeogeographic Implications

**Figure 34:**
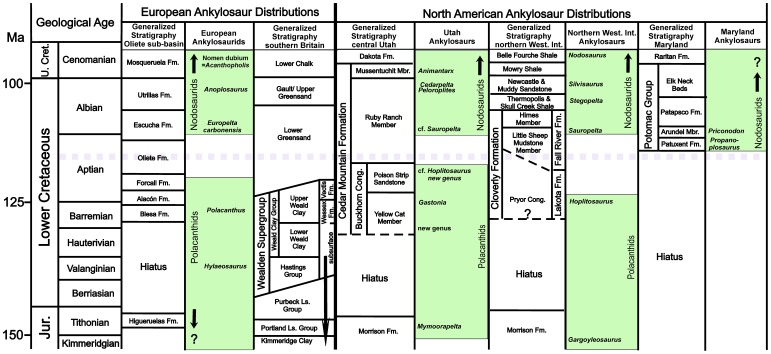
Distribution of polacanthids and nodosaurid ankylosaurs in Europe vs. that of North America. Dashed gray line indicates interval of ankylosaur fauna turnover.

The near simultaneous appearance of nodosaurids in both North America and Europe is worthy of consideration (Fig. 34). *Europelta* is the oldest nodosaurid known in Europe, it derived from strata in the lower Escucha Formation that is dated to early Albian. The oldest nodosaurid from western North America is *Sauropelta*, which in the lower part of its range is in the lower Albian Little Sheep Mudstone Member (B interval) of the Cloverly Formation in northern Wyoming and southern Montana [99], [153] with an ash bed 75 meters above the base near the top of the member providing an age of 108.5±0.2 Ma [154]. Nodosaurid remains from eastern North America appear to be older. Teeth of a large nodosaurid *Priconodon crassus* are known from the Arundel Clay of the Potomac Group [77], [155], which palynology dates as near the Albian-Aptian stage boundary [156]. The hatchling *Propanoplosaurus* is from the base of the underlying Patuxent Formation of the Potomac Group of Maryland, which has been dated as late Aptian [157], [158], making *Propanoplosaurus* the oldest known nodosaurid. Polacanthid ankylosaurs characterize pre-Aptian faunas in both Europe [11], [12], [37]-[39] and North America [70], [95], [159]. We have not been able to document a specific example of *Polacanthus* in the Lower Aptian Vectis Formation of the Wealden Group, although *Polacanthus* has been reported to occur in those strata [10]-[12], [82], [160]. However, polacanthids are present in the lower Aptian Morella Formation of northeastern Spain [40]. Blows [10] illustrated a block with ankylosaur dorsal vertebrae with the uninformative ventral portion of a pelvic shield fragment and noted it as being from Charmouth, suggesting that there were upper Albian polacanthids in England [160]. However, the specimen NMW 92.34G.2 was actually found on the beach further west at Charton Bay and may have come from either the Aptian (Lower Greensand) or Albian (Upper Greensand). Only preparation of the dorsal surface of the pelvic shield would reveal if the specimen is a polacanthid or nodosaurid. A large polacanthid (BYU R254) occurs in the Poison Strip Sandstone Member of the Cedar Mountain Formation [156]. It is not a nodosaurid close to *Sauropelta* as reported by Carpenter and others [97], but a polacanthid that was initially described as cf. *Hoplitosaurus* [161]. These rocks have been dated as lower to middle Aptian by laser ablation of detrital zircons and by U-Pb dating of early diagenetic carbonate [162]. A fragmentary large nodosaurid with massive cervical spikes that may be referred to as cf. *Sauropelta* (DMNS 49764) has been recovered from the overlying Ruby Ranch Member about 20 m up section in the same region [163] in strata interpreted to be of Lower Albian age [162]. Thus, the youngest polacanthids occur in the lower to possibly mid-Aptian and the oldest documented nodosaurids occur in the upper Aptian or lower Albian in both Europe and North America with no discernible stratigraphic overlap (Fig. 34). Why this faunal discontinuity occurs is unknown. There are no documented significant changes in sea level or shifts in geochemical indicators to suggest a geological or environmental change that would affect ankylosaurs on both continents at approximately the same time [164]. However, the OAE1a or “Sella” organic burial episode near the base of the Aptian was followed by a positive carbon isotope excursion that may have precipitated longer-term environmental effects that would result in the turnover of ankylosaurs in the “middle” Aptian [165]. In North America, “medial” grade iguanodonts (basal Steracosterna) are replaced by the considerably more primitive basal iguanodont *Tenontosaurus* at this time, while in Europe the lower Albian more derived iguanodont *Proa* is phylogenetically close to *Iguanodon* [43], [159] at the base of Hadrosauriformes [43], documenting different patterns of faunal change for iguanodonts and ankylosaurs. Therefore, a cause for this faunal turnover, which might specifically have affected ankylosaurs, should be sought. Ankylosaurs are low feeders, so perhaps the rapid ongoing radiation of flowering plants at this time [166]-[170] might have driven their diversification. It has been proposed that this floral revolution was linked to a decline in atmospheric CO2 concentrations [171] or, more likely, an increase in CO2 and global warming resulting from massive early Aptian volcanic activity forming the Ontong Java and Manihiki plateaus [172], [173]-[174]. Therefore the rapid domination of shrubby angiosperms may have caused a disruption in the availability of forage to which polacanthids were adapted. Kirkland and others have proposed that North America became isolated from Europe at the end of the Barremian [159], [175]. Certainly the timing of the appearance of nodosaurids on both continents indicates that the origins of the clade preceded the complete isolation of North America and Europe pushing up this date in to at least the “middle” Aptian. The separation of the Nodosauridae into a North American Nodosaurinae and a European Struthiosaurinae by the end of the Aptian, would thus provide a revised date for the isolation of North America from Europe with rising sealevel.

Additionally, whereas there is no definitive evidence for nodosaurids in Asia, apparently polacanthids entered Asia in the later portion of the Early Cretaceous and survived there in isolation into the early Late Cretaceous.

## Conclusions

*Europelta carbonensis*, a new nodosaurid ankylosaur from the lower Albian Escucha Formation in Spain represents the earliest member of a European clade of nodosaurs defined as the Struthiosaurinae. Other members of this Late Cretaceous clade include: *Anoplosaurus*, *Hungarosaurus*, and *Struthiosaurus*. This clade of nodosaurs replaced the polacanthids in Europe during the Albian, similar to the Albian replacement of polacanthids by nodosaurids in North America.
